# Self‐Doped and Biodegradable Glycosaminoglycan‐PEDOT Conductive Hydrogels Facilitate Electrical Pacing of iPSC‐Derived Cardiomyocytes

**DOI:** 10.1002/adhm.202403995

**Published:** 2025-02-28

**Authors:** Daniel Hachim, Olivia Hernández‐Cruz, James E. J. Foote, Richard Wang, Matthew W. Delahaye, Daniel J. Stuckey, Zhiping Feng, Jonathan P. Wojciechowski, Luke C. B. Salter, Junliang Lin, Sian E. Harding, Molly M. Stevens

**Affiliations:** ^1^ Department of Materials Department of Bioengineering and Institute of Biomedical Engineering Imperial College London Exhibition Road London SW7 2AZ UK; ^2^ Department of Physiology Anatomy and Genetics Department of Engineering Science Kavli Institute for Nanoscience Discovery University of Oxford Sherrington Road Oxford OX1 3QU UK; ^3^ School of Pharmacy Faculty of Chemistry and Pharmacy Pontifical Catholic University of Chile AV. VICUNA MACKENNA 4860 Santiago 7820436 Chile; ^4^ National Heart and Lung Institute Imperial College London Du Cane Road London W12 0NN UK; ^5^ Centre for Advanced Biomedical Imaging University College London 72 Huntley Street London WC1E 6DD UK

**Keywords:** biodegradable, conductive hydrogels, glycosaminoglycan, iPSC‐cardiomyocytes, self‐doping

## Abstract

Conductive polymers hold promise in biomedical applications owing to their distinct conductivity characteristics and unique properties. However, incorporating these polymers into biomaterials poses challenges related to mechanical performance, electrical stability, and biodegradation. This study proposes an injectable hydrogel scaffold composed of a self‐doped conductive polymer, constituted of a sulfated glycosaminoglycan (GAG) with side chains of PEDOT (poly 3,4‐ethylenedioxythiophene). This brush copolymer is synthesized via oxidative polymerization from an EDOT monomer grafted onto the backbone of the sulfated GAG. The GAG backbone offers biodegradability, while sulfate groups act as acidic self‐doping agents. Conductive hydrogels form through oxime crosslinking, initially existing as a liquid mixture that undergoes gelation within the tissue, allowing for injectability. The conductive hydrogels show tunable stiffness and gelation kinetics influenced by both concentration and pH, and exhibit adhesive properties. They showcase dual ionic and electronic conductivity, where sulfate groups in the GAG backbone act as doping moieties, enhancing conductivity and electrical stability. These properties of conductive hydrogels are associated with the facilitation of electrical pacing of iPSC‐cardiomyocytes. Furthermore, hydrogels exhibit biodegradation and show evidence of biocompatibility, highlighting their potential for diverse biomedical applications.

## Graphical abstract

The electrical properties and performance of conductive hydrogels based on sulfated‐glycosaminoglycan‐PEDOT (poly 3,4‐ethylenedioxythiophene) brush copolymers is studied, showing that the higher sulfation degree of heparin confers hydrogels with the highest conductivity. Heparin‐PEDOT hydrogels facilitate the electrical pacing of iPSC‐derived cardiomyocytes, can be injected into the heart and show tunable biodegradation.

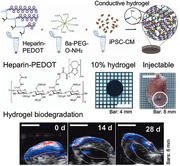

## 1 Introduction

Conductive polymers and their materials are receiving increasing attention in multiple fields, as their electrical conductivity resembles that of metals and inorganic compounds, while possessing flexibility and ease in synthesis, modification, and fabrication.^[^ ^1^ ^]^ These materials also possess unique optical and electronic properties that make them suitable for a range of optoelectronic and sensing applications.^[^ ^1^, ^2^ ^]^ Moreover, materials containing organic semiconductive polymers are sensitive to both electron and ion charge transport, meaning that they are inherently capable of transducing ionic and electric signals in biological systems, while their inorganic counterparts are generally impermeable to ions.^[^ ^1^, ^3^ ^]^ Conductive polymers are for this reason sensitive to electrochemical changes in the environment and morphology of the material, making these materials highly attractive for biomedical applications.

In tissues and organs, numerous functions are regulated by electrical signals, including neuronal synapses, muscle contraction, wound healing, embryonic development and the electromechanical activity of the beating heart.^[^ ^4^ ^]^ Therefore, the use of polymers that are electrically active in tissue engineering scaffolds represents a major opportunity to recapitulate more closely the intrinsic properties of native tissues and therefore enhance functional repair. In this context, there are a number of challenges that need to be addressed before materials made of conductive polymers can be used in clinical settings. Conductive polymers are typically highly conjugated and rigid molecules, resulting in materials with poor mechanical properties – brittle and highly prone to delamination.^[^ ^5^ ^]^ In tissue engineered constructs, the mechanical mismatch between the material and tissue is highly associated to a chronic inflammatory host response and negative regenerative outcomes.^[^ ^6^ ^]^ This challenge has been partially addressed with the fabrication of blends of conductive materials and other scaffold materials with suitable mechanical performance. These include blends of hydrogels with PEDOT:PSS (poly 3,4‐ethylenedioxythiophene: poly 4‐styrenesulfonate) nanoparticles, carbon nanotubes or metal nanoparticles; deposition of conductive films in biocompatible patches and scaffold blends.^[^ ^4^, ^7^ ^]^ Recently, copolymers between mechanically compliant polymers (e.g., polycaprolactone, polyethylene glycol, polyurethane) and oligomers of conductive polymers (e.g., oligoaniline, oligoEDOT) have been synthesized, resulting in mechanically compliant electroactive polymers with superior processability.^[^ ^8^ ^]^ However, the conductivity of these materials is compromised due to the block architecture and/or short length of the conductive moieties in the resulting polymer, disrupting the conduction through the backbone chain and π – π stacking interactions.

Another common challenge of conductive materials is the limited electronic stability in physiological conditions, as they suffer oxidation in contact with body fluids, losing their conductive state irreversibly.^[^ ^9^ ^]^ Oxidation in the polymer chain is a necessary step for electron movement during conduction, but the polymer needs to start from a reduced state to do so or to regenerate its conductive state, a process that is known as doping.^[^ ^9^, ^10^ ^]^ Doping can be induced during fabrication or later by the addition of a doping agent, most of them acids that donate a proton to the conductive polymer structure for electron delocalization. In biological systems, the doping agents diffuse away or lose doping capacity over a short period of time, and hence one strategy to prevent electric deterioration has been immobilization of the dopant within the conductive material.^[^ ^9^, ^11^ ^]^ For example, phytic acid has been immobilized via electrostatic interactions within a chitosan film, that was used as scaffold to grow a polyaniline (PANI) layer, approach that delayed the deterioration of electrical properties in the material.^[^ ^9c^ ^]^ Regardless, the deterioration of the electrical properties was directly attributed to the leakage of doping agent. Leakage should be easily prevented by covalently immobilizing the doping agent, either by functionalization of the doping agent or incorporation of a doping domain in the polymer structure. Of note, only a few doping agents have been shown to be noncytotoxic, while most efficient organic acids are not compatible with biological systems at any concentration.^[^ ^9^, ^10^, ^12^ ^]^

Most fabrication methods for conductive materials are performed in conditions that are not compatible with living systems, due to the presence of hazardous reagents and initiators.^[^ ^4^, ^13^ ^]^ For that reason, most of these materials are used with cells and tissues after fabrication and purification, as encapsulation of cells and injectability is not possible. Similarly, the use of conductive materials as tissue engineered scaffolds will not reach its full potential until they present biodegradability. An optimal tissue engineering approach seeks to provide a transient supporting matrix that restores the lost structure and function of a tissue, and to do so the scaffold needs to degrade and be replaced by healthy tissue.^[^ ^14^ ^]^ To the best of our knowledge, conductive scaffolds with full length chains of conjugated polymers have not yet achieved biodegradation. Most strategies to provide conductive materials with biodegradation rely in blends containing biodegradable polymers^[^ ^15^ ^]^ or by erosion mechanisms,^[^ ^16^ ^]^ in which the conjugated polymers do not truly degrade, and could accumulate in tissues and organs. Another strategy is the grafting of conjugated oligomers in biodegradable polymers, which resulted in compromised conductivity compared to their fully polymerized conjugated versions, but highly attractive for biomedical applications where their electroactive properties produce the intended response (e.g., drug release).^[^ ^5^, ^8^, ^17^ ^]^ Despite not reporting electrical properties, one interesting approach consisting of a thiophene‐imidazole conjugated copolymer used for fabricating highly fluorescent nanoparticles, showing biodegradation under the presence of reactive oxygen species (ROS) produced by macrophages in culture.^[^ ^18^ ^]^ Considering these points, an ideal conductive material for tissue engineering applications should consist of a polymer with biodegradable, mechanically compliant and stable electronic properties. A desirable but optional feature is minimally invasive implantation. In the heart, current conductive scaffolds would require high risk and complex surgical procedures (e.g., open chest surgery), but new developments in injectable and shear‐resistant scaffolds opened the possibilities toward minimally invasive implantation via injection, catheters and laparoscopic routes.^[^ ^19^ ^]^

Here, we developed an injectable hydrogel scaffold composed of a biodegradable and self‐doped conductive polymer. We hypothesized that the self‐doping capacity and biodegradability can be obtained by synthesizing a brush copolymer composed of a sulfated glycosaminoglycan (GAG) backbone and side chains of PEDOT. Self‐doping capacity was provided by the acidic sulfate groups in the glycosaminoglycan backbone chains, such as heparin and chondroitin sulfate, which are able to donate protons to their PEDOT side chains, promoting the formation of a conduction band. The doping capacity of heparin has been previously reported to increase the electrical stability of PANI in hydrogel blends and PEDOT films.^[^ ^7^, ^12^, ^20^ ^]^ GAGs are naturally biodegraded or depolymerized by either eliminative cleavage of lyases (e.g., heparin lyase) or by endohydrolytic cleavage of hydrolases (e.g., heparinase I, chondroitinase ABC), often specific for each specific GAG type or modified residues in the polysaccharide.^[^ ^21^ ^]^ Degradation of the GAG backbone of the copolymer would hence generate small soluble fragments that diffuse away in body fluids and later be excreted. Hydrogel formation and injectability is based on the formation of oximes as the crosslinking mechanism. Oximes are a type of reversible crosslinking that is formed by the reaction of an amino‐oxy group with aldehydes and ketones.^[^ ^22^ ^]^ Higher degree of reversibility in the reaction is obtained using aldehydes, while the equilibrium with ketones tends to go toward the oxime products, showing less reversibility and more stable hydrogels. Both reaction equilibriums can be modified with pH, hence hydrogel gelation kinetics and reversibility can be tuned with pH. Previous examples of these types of hydrogels using PEG systems have demonstrated feasibility of hydrogel injection via catheter, with lower pH accelerating gelation kinetics.^[^ ^22d^ ^]^

## 2 Results and Discussion

### 2.1 Synthesis of Glycosaminoglycan‐PEDOT Polymers

The conductive copolymers were synthesized using three distinct glycosaminoglycans (GAGs) with different degree of sulfation. Heparin (Hep) has been chosen as a backbone polymer with high degree of sulfation, having two to three sulfate groups per disaccharide unit. Chondroitin sulfate A (CS) is used as a polymer backbone with low degree of sulfation, with one sulfate group per disaccharide unit. Hyaluronic acid (HA) has no sulfation and hence will be considered as a control.

**Figure 1:**
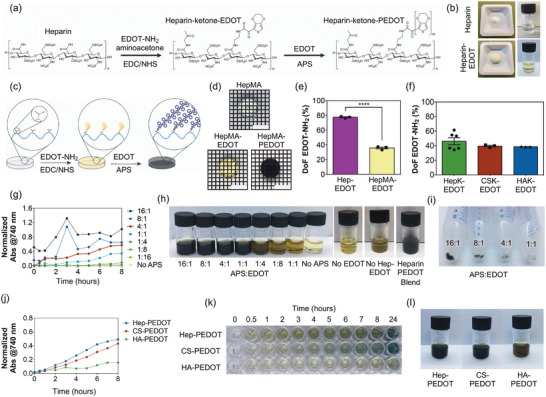
a) Schematic of EDOT‐NH_2_ monomer grafting followed by PEDOT side chain polymerization on glycosaminoglycans, in this example heparin. b) Solid appearance and solubility in water of Hep‐EDOT and heparin. c) Schematic of in situ EDOT‐NH_2_ grafting followed by PEDOT side chain polymerization on preformed GAG‐hydrogels. d) Representative image of a 10% HepMA, HepMA‐EDOT and HepMA‐PEDOT hydrogels obtained via in situ grafting and polymerization. Scale bars represent 4 mm. e) Degree of functionalization of EDOT‐NH_2_ monomer on heparin in solution (Hep‐EDOT) and in situ on heparin hydrogels (HepMA‐EDOT), using UV‐Vis quantification at 320 nm. Bars represent the mean of three samples (*N* = 3) ± SEM. Statistical significance with (****) *p* < 0.0001, using two‐tailed t tests. f) Degree of functionalization of EDOT‐NH_2_ monomer while grafting both monomer and aminoacetone at the same time on heparin (HepK‐EDOT), chondroitin sulfate (CSK‐PEDOT) and hyaluronic acid (HAK‐PEDOT), using UV‐Vis quantification at 320 nm. Bars represent the mean of three samples (*N* = 3 – 6) ± SEM. No statistical significance was detected, using one‐way ANOVA. g) Kinetics of PEDOT growth polymerization as normalized absorbance at 740 nm, using different APS to EDOT ratios. Plot shows representative curve of three samples (*N* = 3). h) Side chain growth polymerization crude after 24 h, using different APS to EDOT ratios and control reactions in absence of EDOT or Hep‐EDOT, as well as a reaction mixture of both heparin and EDOT. i) Insoluble PEDOT side products from side chain polymerization, using different APS to EDOT ratios, precipitated by centrifugation. j) Kinetics of PEDOT growth polymerization as normalized absorbance at 740 nm, starting from Hep‐EDOT, CS‐EDOT, and HA‐EDOT. Plot shows representative curve of three samples (*N* = 3). k) Side chain growth polymerization crude reactions over time and after 24 h l), starting from Hep‐EDOT, CS‐EDOT, and HA‐EDOT.

The solubility of both GAGs and poly(3,4‐ethylenedioxythiophene) (PEDOT) is limited in organic solvents and hence direct grafting is not possible. GAGs are generally soluble in aqueous solvents, while PEDOT solubility in both aqueous and organic solvents is extremely low. PEDOT is generally obtained via polymerization from the more soluble monomer EDOT, using chemical or electrochemical methods.^[^ ^23^ ^]^ Therefore, GAG‐PEDOT copolymers have been synthesized in two steps – starting with the coupling of an in‐house made EDOT‐NH_2_ monomer into the lateral carboxylic acid groups of the GAG, followed by PEDOT side chain polymerization, in aqueous conditions (**Figure** **1**a). Alternatively, monomer grafting and PEDOT polymerization can be performed in situ on glycosaminoglycan‐hydrogels, and potentially on any other carboxylic acid containing material (Figure 1c).

Synthesis of the EDOT‐NH_2_ monomer occurs via glyoxylation of EDOT and coupling of a Boc‐protected ethylene diamine, as previously described,^[^ ^24^ ^]^ then followed by acid deprotection and purification. The resulting monomer was observed to be a water‐soluble yellow powder, with a yield of 75%. NMR was used to validate the structure and purity, which can be found in Figure S1 (Supporting Information). Initial optimization of the polymer synthesis and subsequent fabrication was done using heparin. EDOT‐NH_2_ was grafted into the carboxylic acid residues of the GAG backbone chain using EDC/NHS coupling. During this step, the crosslinker amino‐acetone can also be added to the polymer for subsequent hydrogel fabrication. ATR‐FTIR revealed successful formation of an amide bond between heparin and EDOT‐NH_2_, as revealed in peaks at 1475 cm^−1^ (amide C─N stretching) and 1675 cm^−1^ (amide C═O stretching) (Figure S2a, Supporting Information), while quantification via UV–Vis at 320 nm showed that 1.4 × 10^−4^ mmol of EDOT‐NH_2_ were bound per mg of heparin, which corresponds to a degree of functionalization (DoF) of 80% (Figure 1e). The success of the grafting was also visually evident on to the coloration of Hep‐EDOT, which after freeze drying results in a yellow spongy material, soluble in water (Figure 1b). Normal heparin is white and transparent once is solubilized in water. This was in agreement with ^1^H NMR results, as shown in Figure S2b (Supporting Information): δ 7.75 (aryl‐proton) and δ 2.1–2.5 (–CH_2_– protons) peaks from EDOT‐NH_2_ monomer. When amino‐acetone was added at the same time with EDOT‐NH_2_, the DoF decreased to 46%, which is expected as both species are competing for the same carboxylate group in the heparin backbone, to obtain heparin‐ketone‐EDOT (HepK‐EDOT). Results from FTIR and ^1^H NMR spectra are in agreement with the successful grafting of both EDOT‐NH_2_ and amino‐acetone, this last one with a DoF of 28% (Figure S3, Supporting Information). The grafting in both chondroitin sulfate and hyaluronic acid was successful and show similar EDOT monomer grafting of 40% and 39%, respectively (Figure 1f). In preformed heparin‐methacrylate (HepMA) hydrogels, a yellow coloration is retained after the reaction, in which quantification revealed that 6.3 × 10^−5^ mmol of EDOT‐NH_2_ were grafted into the hydrogel, which is equal to a DoF of 36% (Figure 1e). Lower DoF of EDOT‐NH_2_ on HepMA is likely due to the grafting occurring to an already established network of heparin‐methacrylate polymer in the hydrogel. When EDC/NHS is not added into the reaction mixture, only a weak yellow background coloration is observed, likely due to electrostatic interactions between protonated amino groups in EDOT‐NH_2_ and anionic groups in heparin.

Hep‐PEDOT is obtained from an aqueous radical side‐chain polymerization, growing from the grafted EDOT‐NH_2_ in the heparin backbone, by adding EDOT monomer in presence of ammonium persulfate (APS), the radical initiator. An optimal initiator to monomer ratio ensures that the reaction kinetics further enhances the formation of a soluble heparin‐PEDOT product, with a minimal production of free insoluble PEDOT impurities, which are easily removed by filtration. Therefore, different APS:EDOT ratios were tested to study the polymerization kinetics and formation of the main Hep‐PEDOT product and side products. Polymerization kinetics and product formation were assessed via UV‐Vis spectroscopy following the change in absorbance at 740 nm (Figure 1g). Of note, the course of the reaction can be easily tracked by the changes in the coloration of an initial yellow Hep‐EDOT solution, toward a darker yellow/brown and then dark blue, to finish with a fully polymerized black solution of Hep‐PEDOT.

Kinetics curves revealed that APS:EDOT ratios equal or below to 1:4 are not enough to start the polymerization process, being quite similar in absorbance to the no‐APS control (Figure 1g,h), while coloration showed dark‐yellow to brown which indicated little to no PEDOT formation. Results also showed that increasing the APS:EDOT ratio accelerated the polymerization kinetics and the amount of product up to a certain point. The high variability and inconsistency observed in the 16:1 and 8:1 ratios (Figure 1g) suggested that the formation of insoluble free PEDOT is larger than at lower ratios, which is confirmed by the significantly larger pellet formed after centrifugation of the reaction mix (Figure 1i). The presence of insoluble PEDOT particles produces light scattering and disrupts the absorbance measurement. On that note, the 1:1 ratio produces almost no insoluble product, but the polymerization does not seem to have reached completion. Overall, the results showed that the optimal APS:EDOT ratio of 4:1 results in the highest Hep‐PEDOT yield with minimal formation of side products, which can be removed via filtration.

Additional controls using this ratio were tested to further confirm that polymerization occurs from the grafted EDOT‐NH_2_ in the heparin backbone. The first observation is that polymerization did not occur when the free EDOT monomer is not present, and the initial Hep‐EDOT does not polymerize by itself in presence of APS (Figure 1h). The second control shows that polymerization is not complete when free EDOT monomers and APS are mixed, demonstrating that the polymerization involves both grafted EDOT and the free EDOT in solution. Because EDOT‐NH_2_ has only one end available for polymerization, the only possible polymerization mechanism is via side‐chain growth. Therefore, the 4:1 APS to EDOT ratio has been chosen for the rest of the study and for the synthesis of all other GAG‐PEDOT polymers. Interestingly, the polymerization kinetics on all three GAGs revealed their sulfation degree also influences the polymerization rate and degree of completion. The polymerization rate is faster in heparin (the highest sulfation degree), and slower in chondroitin sulfate (lower sulfation degree). In both cases they reached completion at 24 h – no changes in absorbance and coloration of the reaction mixtures occurred after that. On the other hand, hyaluronic acid, which has no sulfation, resulted in the slowest rate of polymerization and did not reach completion (Figure 1j,k,l). This confirms that the polymerization requires the presence of sulfates in the polymer to reach completion, suggesting they act as acid self‐doping agents, in a similar manner polystyrene sulfonate (PSS) acts as an acidic doping agent during PEDOT:PSS particle synthesis.^[^ ^25^ ^]^ The final purified and freeze dried Hep‐PEDOT product is a black spongy material, soluble in water, resulting in black‐translucent solutions that do not precipitate over time. Contrarily, controls where PEDOT was polymerized in presence of heparin (heparin and PEDOT blend), resulted in turbid solutions that precipitated over time, which is proper of particle suspensions. In HepMA hydrogels, the in situ polymerization of PEDOT was also confirmed by the hydrogel turning translucent black (Figure 1d).

### 2.2 Fabrication of Conductive GAG‐PEDOT Oxime Hydrogels

**Figure 2:**
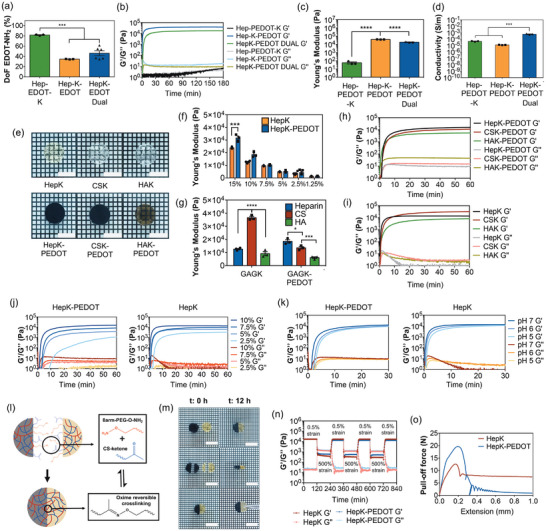
a) Degree of functionalization of EDOT‐NH_2_ monomer on heparin when grafting is performed as first step (Hep‐EDOT‐K), performed after grafting of aminoacetone (Hep‐K‐EDOT) and performed at the same time (HepK‐EDOT Dual). Bars represent the mean (*N* = 3 – 6) ± SEM. Statistical significance with (***) *p* < 0.001, using one‐way ANOVA with Tukey post‐tests. b) Gelation kinetics of Hep‐PEDOT‐K, Hep‐K‐PEDOT and HepK‐PEDOT Dual hydrogels. Curves represent the mean of three samples (*N* = 3). c) Stiffness (Young's modulus) of Hep‐PEDOT‐K, Hep‐K‐PEDOT and HepK‐PEDOT Dual hydrogels. Bars represent the mean of three samples (*N* = 3) ± SEM. Statistical significance with (****) *p* < 0.0001, using one‐way ANOVA with Tukey post‐tests. d) Conductivity of Hep‐PEDOT‐K, Hep‐K‐PEDOT and HepK‐PEDOT Dual hydrogels. Bars represent the mean of three samples (*N* = 3) ± SEM. Statistical significance with (****) *p* < 0.0001, using one‐way ANOVA with Tukey post‐tests. e) Representative images of nonconductive GAG hydrogel controls (HepK, CSK, and HAK) and conductive GAG‐based hydrogels (HepK‐PEDOT, CSK‐PEDOT, and HAK‐PEDOT). Scale bars represent 4 mm. f) Stiffness (Young's modulus) of both HepK and HepK‐PEDOT hydrogels at different total mass concentrations. Bars represent the mean (*N* = 3–5) ± SEM. Statistical significance with (****) *p* < 0.0001, using two‐way ANOVA and Sidak's post‐tests to compare between HepK and HepK‐PEDOT. g) Stiffness (Young's modulus) of HepK‐PEDOT, CSK‐PEDOT, and HAK‐PEDOT hydrogels at 10% total mass concentration. Bars represent the mean (*N* = 3–4) ± SEM. Statistical significance with (*) *p* < 0.05, (***) *p* < 0.001, and (****) *p* < 0.0001, using one‐way ANOVA and Tukey post‐tests to compare within nonconductive and conductive hydrogel groups. Gelation kinetics of h) nonconductive hydrogel controls based on heparin (HepK), chondroitin sulfate (CSK) and hyaluronic acid (HAK) and i) conductive hydrogels based on heparin (HepK‐PEDOT), chondroitin sulfate (CSK‐PEDOT) and hyaluronic acid (HAK‐PEDOT). Curves represent the mean of (*N* = 3–4). Effect of j) total mass concentration and k) pH in the gelation kinetics of HepK‐PEDOT and HepK hydrogels. Curves represent the mean (*N* = 3–5). l) Schematic of hydrogel self‐healing mechanism and oxime reversible crosslinking. m) Example of self‐healing properties of the oxime hydrogels by cutting and putting together two 10% hydrogel pieces of HepK and HepK‐PEDOT, then manually inspected at 12 h. Scale bars represent 6 mm. n) Rheological assessment of self‐healing properties of HepK and HepK‐PEDOT hydrogels. Hydrogel rupture is performed at 500% strain force, which is confirmed by inversion of the G’ and G’’ curves and left to recover at 1% strain force. Three cycles of rupture and healing of hydrogels are shown. Curves represent the mean of three samples (*N* = 3). o) Adhesiveness of HepK and HepK‐PEDOT hydrogels via pull‐out testing. Plot shows the representative curve of three samples (*N* = 3).

Due to the good solubility of all glycosaminoglycan‐PEDOT in aqueous solutions, the presence of a crosslinker is required to form a hydrogel scaffold. To render conductive hydrogels injectable, we have chosen oxime formation as a crosslinking mechanism, which is the product of a reaction between one molecule containing ketone groups and another one containing amino‐oxy groups, releasing water as a side product, being highly cyto‐ and bio‐ compatible.^[^ ^22^ ^]^ An 8‐arm amino‐oxy‐PEG was synthesized, adapted from a previously described synthesis of a 4‐arm amino‐oxy‐PEG.^[^ ^22d^ ^]^ The ketone crosslinker (aminoacetone) was incorporated into the GAG‐PEDOT polymer, and three different grafting strategies were assessed on heparin to obtain a hydrogel with optimal gelation kinetics, mechanical properties and conductivity. These included 1) Hep‐PEDOT‐K: aminoacetone conjugation after Hep‐PEDOT polymerization, 2) Hep‐K‐PEDOT: aminoacetone conjugation before EDOT‐NH_2_ grafting into heparin, and 3) HepK‐PEDOT Dual: one‐step aminoacetone and EDOT‐NH_2_ grafting into heparin. A degree of functionalization (DoF) of 80% is obtained when EDOT is grafted in the first step (**Figure** **2**a), and significantly diminished to 35% when grafted after aminoacetone – likely a consequence of presenting less carboxylic acid sites for grafting. When both EDOT and aminoacetone are grafted in a single reaction, there is competition for carboxylic acids and EDOT grafting goes down to 46%. The impact of these changes in grafting on gelation kinetics and mechanical properties of the resulting hydrogels were studied with rheological testing. Results revealed hydrogel formation in all copolymers, however, conjugating the aminoacetone crosslinker after the synthesis of Hep‐EDOT results in a hydrogel with significantly slower gelation kinetics (>120 min) and poor stiffness (plateau G' = 58 Pa) (Figure 2b,c). This is likely due to the lower number of free carboxylic acid groups available after EDOT grafting. As it could be expected, conjugating aminoacetone before grafting EDOT‐NH_2_ results in hydrogels with the fastest gelation kinetics (≈5 min, fully crosslinked by 15 min) and highest stiffness (plateau G' = 39 000 Pa). When conjugation is performed at the same time (HepK‐PEDOT Dual), gelation kinetics is slower than Hep‐K‐PEDOT, gelation occurred within 10 min after mixing, reaching complete crosslinking by 30 min. There are no significant differences in stiffness compared to Hep‐K‐PEDOT hydrogels, showing a value of plateau *G*' = 18500 Pa.

Conductivity of these hydrogels was assessed via four‐point probe measurements. Before doing so, the polymers were treated in an ion exchange column to ensure complete re‐protonation of sulfonates in the polymers and full doping capacity. Results using both treatments showed consistently that the highest conductivity in the hydrogel is obtained when using HepK‐PEDOT Dual copolymer (Figure 2d). Even though grafting the EDOT‐NH_2_ before aminoacetone ensures the highest DoF (80%), conductivity in Hep‐PEDOT‐K was not the highest, suggesting that other effects in the hydrogel matrix (e.g., crosslinking density) are playing a role. Therefore, the most appropriate strategy to synthetize the polymer is dual grafting of both EDOT‐NH_2_ and the aminoacetone crosslinker, followed by PEDOT side chain polymerization (HepK‐PEDOT Dual), which offers an appropriate gelation time for hydrogel injection and gelation (10 min), strong but soft mechanical stiffness (in the kPa range) and the highest conductivity of all tested copolymers. Hereafter, this copolymer will be referred as HepK‐PEDOT, while the nonconductive control heparin hydrogel, HepK, does not possess PEDOT in the polymer structure.

### 2.3 Mechanical Performance of Conductive GAG‐PEDOT Oxime Hydrogels

All studied glycosaminoglycan‐based oxime hydrogels are soft, translucent and their stiffness can be tuned with concentration (Figure 2e). In general, the presence of PEDOT in the copolymer did not have a significant impact in the mechanical properties of the hydrogels. A higher stiffness is observed at 15% (w/v), which is likely due to the higher density of PEDOT in the hydrogel matrix (Figure 2f). Regardless, both hydrogels are similar and within the same 10^4^ Pa magnitude, matching the stiffness of heart muscle.^[^ ^26^ ^]^ Similarly, the stiffness of chondroitin sulfate (CSK/CSK‐PEDOT) and hyaluronic acid (HAK/HAK‐PEDOT) hydrogels (10% (w/v)) is also in the 10^4^ Pa magnitude (Figure 2g), with some differences in stiffness that did not show correlation between conductive and nonconductive hydrogels. Gelation kinetics showed to be quite similar between all GAG‐based hydrogels (Figure 2h,i).

Moreover, the concentration of hydrogels influenced the gelation rate, with lower concentrations exhibiting delayed gelation times in both HepK and HepK‐PEDOT hydrogels (Figure 2j). This is likely an expected consequence of having lower crosslinking density. Similarly, the pH of the solution changes gelation kinetics, with results showing that acidic pH delayed the gelation time in both oxime hydrogels but did not change stiffness as concentration does (Figure 2k). The responsiveness to pH is due to the oxime crosslinking, in which the equilibrium between reactants (ketones and amino‐oxy species) and products (oximes) responds to changes in environmental pH – equilibrium that predominates toward the products when the pH is neutral but can be reversed toward the reactants in acidic environment (Figure 2l). This responsiveness may present clinical relevance, being able to delay the gelation rate in scenarios where surgeons would require additional time for hydrogel injection. Interestingly, crosslinking reversibility provides conductive hydrogels with self‐healing properties. Self‐healing was tested by cutting in half a cylindrical conductive hydrogel and a nonconductive hydrogel, then attaching one half of each hydrogel to the other, followed by assessment of hydrogel bonding over time. Bonding occurs within 6 h, resulting in a single cylindrical piece of hydrogel that does not break under mechanical manipulation and inspection (Figure 2m). Regardless of the tight bonding between both hydrogel pieces, the polymers remained highly confined on each side of the whole structure, with no polymer intertwining over time. Self‐healing was also demonstrated via rheological assessments. The structure of the conductive HepK‐PEDOT hydrogel and the HepK hydrogel controls were disrupted using high strain forces (500%), which is confirmed when the storage modulus (G') and loss modulus (G″″) become inverted. Once the strain forces come back to basal levels (0.5%), the storage and loss modulus are restored to their original values. This cycle was repeated several times and the hydrogels always recovered their original mechanical properties after disruption (Figure 2n), which can only happen in self‐healing materials.

We observed that both conductive and nonconductive oxime hydrogels exhibited some degree of adhesiveness, hence we studied adhesiveness via standard pull‐out testing. To do so, we sandwich‐casted the hydrogels between two glass slides and performed uni‐axial pull‐out, as shown in Figure 2o. Results showed that the adhesive strength of the conductive HepK‐PEDOT hydrogels was 31.6 kPa (maximum force 19.7 N with 0.2 mm of displacement), while in nonconductive HepK hydrogels was 20.2 kPa (maximum force 12.6 N with 0.17 mm of displacement).

### 2.4 Electronic Properties of Conductive GAG‐PEDOT Oxime Hydrogels

Conductive materials made of synthetic conjugated polymers like PEDOT provide conductivity to materials via electron transport through their conjugated chains and π‐π stacking interactions.^[^ ^27^ ^]^ In glycosaminoglycan hydrogels, rich in polyanion species, the presence of ions in the liquid phase generates ionic conductivity.^[^ ^28^ ^]^ In living systems, conductivity among cells and tissues is propagated through ion channels.^[^ ^29^ ^]^ It is hence expected that the conductivity of GAG‐PEDOT hydrogels originates from both electrons from PEDOT and ions in the GAG chain. To elucidate the contributions of electronic and ionic conductivity, the conductivity of hydrogels was measured in a four‐point probe (4PP) in hydrated and dry state (after freeze drying). Hydrated hydrogels account for the total of both electronic and ionic conductivity, while in dry state the flow of ions is negligible, hence being equal to the electronic conductivity. In general, ion mobility is only possible in liquid or semisolid phases and restricted in the solid phase. Therefore, the ionic conductivity is the difference between the total (hydrated) and electronic (dry) conductivity.

**Figure 3:**
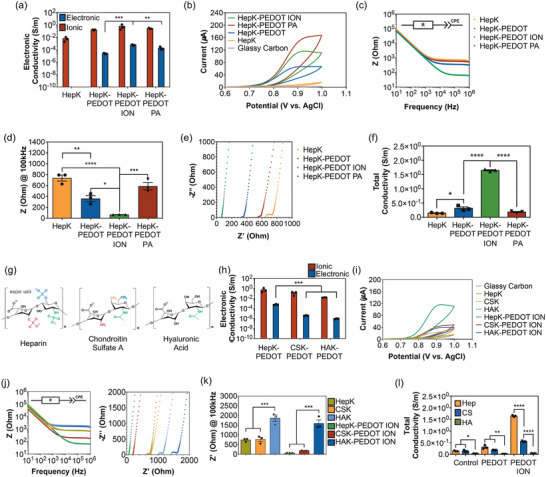
a) Ionic and electronic conductivity of nonconductive heparin hydrogel (HepK), basal conductive hydrogel (HepK‐PEDOT), conductive hydrogels treated using polymer treated with ion exchange columns (HepK‐PEDOT ION) and conductive hydrogels with polymers treated with phytic acid (HepK‐PEDOT PA). Bars represent the mean of three samples (*N* = 3) ± SEM. Statistical significance with (**) *p* < 0.01 and (***) *p* < 0.001, using one‐way ANOVA with Tukey post‐tests to compare the means of either ionic or electronic conductivity. b) Cyclic voltammograms (CV) at 50 mV s^−1^ of HepK, HepK‐PEDOT, HepK‐PEDOT ION and HepK‐PEDOT PA hydrogels. Curves are the representative sample from N = 3. (c) Bode plot showing impedance (Z) at different frequencies for HepK, HepK‐PEDOT, HepK‐PEDOT ION and HepK‐PEDOT PA hydrogels. Curves represent the mean of three samples (*N* = 3). d) Impedance (*Z*) at 100 kHz for HepK, HepK‐PEDOT, HepK‐PEDOT ION, and HepK‐PEDOT PA hydrogels. Bars represent the mean of three samples (*N* = 3) ± SEM. Statistical significance with (**) *p* < 0.01, (***) *p* < 0.001 and (****) *p* < 0.0001, using one‐way ANOVA with Tukey post‐tests. e) Nyquist plots showing the real (Z’) versus the imaginary (Z’’) impedance for HepK, HepK‐PEDOT, HepK‐PEDOT ION and HepK‐PEDOT PA hydrogels. Curves represent the mean of three samples (*N* = 3). f) Total conductivity calculated from circuit fitting of HepK, HepK‐PEDOT, HepK‐PEDOT ION and HepK‐PEDOT PA hydrogels. Bars represent the mean of three samples (*N* = 3) ± SEM. Statistical significance with (*) *p* < 0.05 and (****) *p* < 0.0001, using one‐way ANOVA with Tukey post‐tests. g) Schematics of the structure of heparin, chondroitin sulfate A and hyaluronic acid, used in this study. h) Ionic and electronic conductivity of HepK‐PEDOT, CSK‐PEDOT, and HAK‐PEDOT. Bars represent the mean of three samples (*N* = 3) ± SEM. Statistical significance with (***) *p* < 0.001, using one‐way ANOVA with Tukey post‐tests to compare the means of either ionic or electron conductivity. i) Cyclic voltammograms (CV) at 50 mV s^−1^ of HepK, CSK, HAK, HepK‐PEDOT, CSK‐PEDOT and HAK‐PEDOT hydrogels. Curves are the representative sample from *N* = 3. j) Bode and Nyquist plots of HepK, CSK, HAK, HepK‐PEDOT, CSK‐PEDOT, and HAK‐PEDOT hydrogels. Curves represent the mean of three samples (*N* = 3). k) Impedance (*Z*) at 100 kHz of HepK, CSK, HAK, HepK‐PEDOT, CSK‐PEDOT and HAK‐PEDOT hydrogels. Bars represent the mean of three samples (*N* = 3) ± SEM. Statistical significance with (***) *p* < 0.001, using one‐way ANOVA with Tukey post‐tests. l) Total conductivity calculated from circuit fitting of HepK, CSK, HAK, HepK‐PEDOT, CSK‐PEDOT and HAK‐PEDOT hydrogels. Bars represent the mean of three samples (*N* = 3) ± SEM. Statistical significance with (*) *p* < 0.05, (**) *p* < 0.01 and (****) *p* < 0.0001, using one‐way ANOVA with Tukey post‐tests.

Hydrogel conductivity was measured using the polymer with no treatment (HepK‐PEDOT), treated with an ion exchange column for full sulfate re‐protonation (HepK‐PEDOT ION) or treated with phytic acid (PA). HepK hydrogels were used as controls. Electronic conductivity was observed in all Hep‐PEDOT hydrogels, regardless of treatment, while no electronic conductivity was detected in HepK hydrogel controls (**Figure** **3**a). Ionic conductivity was found to be the predominant contribution to the conductivity of all hydrogels, with at least two orders of magnitude higher than electronic conductivity. No difference in ionic conductivity was found among samples (0.16–0.59 S m^−1^) using this technique. Conversely, the electronic conductivity of the base HepK‐PEDOT hydrogel (10^−5^ S m^−1^) is enhanced when the polymer is re‐protonated, regardless of the treatment, reaching values in the range of 10^−4^ S m^−1^.

The magnitude of current flow measured via cyclic voltammetry (CV) is mostly correlated with the observed differences in electron conductivity. Only samples containing HepK‐PEDOT, regardless treatment, showed an oxidation peak, while HepK control did not show any (Figure 3b). The areas under the curve in HepK‐PEDOT ION hydrogels were larger than in basal HepK‐PEDOT hydrogels, suggesting that the electrochemical activity and current exchange ability of fully reprotonated polymer is higher than the base polymer. HepK hydrogel controls displayed the lowest areas under the curve.

Electrochemical impedance spectroscopy (EIS) was performed on hydrogels sandwich‐casted between two carbon electrodes, and results were consistent with the results from 4PP and CV. The impedance (a measure of resistivity in function of frequency, inverse of conductivity) of basal HepK‐PEDOT hydrogels decreased with increasing frequencies and becomes independent approximately above 10^4^ Hz, while in fully protonated HepK‐PEDOT ION hydrogels independence is observed approximately above 10^5^ Hz (Figure 3c). This response to frequency is typical of PEDOT‐based materials.^[^ ^30^ ^]^ The impedance of nonconductive HepK hydrogels became independent above ≈5 × 10^3^ Hz. The magnitude of the impedance at 10^5^ Hz is lower in HepK‐PEDOT ION hydrogels (68 Ω), followed by the basal HepK‐PEDOT (362 Ω). HepK hydrogels showed the highest impedance (739 Ω) (Figure 3d).

The Nyquist plot (Figure 3e) showed that the correlation between real (Z') and imaginary (Z″″) components in the impedance is linear with angles close to 45°, suggesting the presence of a constant phase element. The impedance at the x‐axis intercept (Z″″ = 0) is entirely real and therefore the Z' represents a resistance. Only HepK hydrogels exhibit a semicircular curve at high frequencies, which is usually present when the resistance to charge transfer is higher. The conductivity values were extrapolated from the resistance obtained from circuit fitting using a resistor and a constant phase element in series. High slopes in all curves indicate that the contribution of Warburg diffusion elements is low and in fact in this case did not fit in our models. The results show that the conductivity of HepK‐PEDOT hydrogels is 0.31 S m^−1^, while the conductivity of HepK‐PEDOT ION hydrogels containing fully protonated polymers increases to 1.65 S m^−1^. Nonconductive HepK hydrogels have significantly lower conductivity, with values of 0.15 S m^−1^, which corresponds to ionic conductivity generated from the sulfated heparin polymer chains. Doping with PA did not increase the total conductivity of HepK‐PEDOT hydrogels, and then only heparin seems to act as a doping agent to increase conductivity, as seen in four‐point probe measurements (Figure 3f).

The influence of sulfation in the polymer has been studied using GAG with distinct sulfation degree. Heparin (Hep) has been used as the backbone chain with the highest degree of sulfation, containing two to three sulfate groups per disaccharide unit.^[^ ^31^ ^]^ Chondroitin sulfate A (CS) was chosen as backbone chain with a low degree of sulfation, containing one sulfate group per disaccharide unit.^[^ ^31^, ^32^ ^]^ Hyaluronic acid (HA) does not possess sulfate groups and hence has been used as polymer backbone control.^[^ ^31^, ^33^ ^]^ The structures of these GAGs are depicted in Figure 3g.

Four‐point probe measurements showed no significant differences in ionic conductivity among GAGs in hydrogels with both untreated and ion‐exchange column treated polymers (Figure 3h). Interestingly, the electronic conductivity in untreated and fully re‐protonated hydrogels increased with a higher degree of sulfation, being HepK‐PEDOT the hydrogel with the highest electronic conductivity, followed by CSK‐PEDOT. Full re‐protonation in both sulfated GAGs, via ion exchange columns, significantly increased the electronic conductivity of the hydrogels, higher in magnitude for HepK‐PEDOT. Ion exchange column treatment of HAK‐PEDOT did not change the conductivity of the hydrogel, which would be expected due to the absence of sulfate groups in the polymer. These results show that sulfates in the backbone polymer donate protons to the PEDOT side‐chain moieties to promote electron delocalization and increase hydrogel conductivity, hence acting as self‐doping polymers. In the absence of sulfates in the structure, doping of PEDOT sidechains does not occur and conductivity is significantly lower.

We continued studying the effects of sulfation degree on fully re‐protonated polymers, for more accurate comparisons. CV assessment shows the oxidation peak in all polymers containing PEDOT, which are not present in either HepK, CSK and HAK controls (Figure 3i). The current exchange in these hydrogels, as seen in the magnitude of their areas under the curve, was shown to be correlated with the degree of sulfation. KepK‐PEDOT ION exhibited the highest current exchange and electrochemical activity, followed by CSK‐PEDOT ION and then HAK‐PEDOT ION.

Similarly, EIS assessments are in agreement with 4PP and CV results. The impedance of all GAG‐PEDOT hydrogels decreased with increasing frequency but became independent at higher frequencies with a higher sulfation degree: HepK‐PEDOT above 10^5^ Hz, CSK‐PEDOT above 10^4^ Hz and HAK‐PEDOT above 10^3^ Hz (Figure 3j). The impedance of nonconductive GAG hydrogels became independent at lower frequencies than their conductive hydrogel variants. The impedance of conductive hydrogels at 10^5^ Hz was found to be lower with higher degree of sulfation. HepK‐PEDOT ION, having the highest degree of sulfation, presents the lowest impedance (68 Ω), followed by CSK‐PEDOT ION (194 Ω) with low sulfation degree (Figure 3k). HAK‐PEDOT ION showed high impedance values (1594 Ω), similar to the values found for HepK (739 Ω), CSK (765 Ω), and HAK (1860 Ω) hydrogel controls, which is expected as no sulfates are present in the backbone of the polymer.

Similar to HepK‐PEDOT hydrogels, the Nyquist plots show that all hydrogels possessed a resistor and constant phase element configuration in series, with differences in the magnitude of resistivity. For example, HAK‐PEDOT and all nonconductive hydrogel controls showed intercepts with significantly higher values in impedance, but also the presence of semicircular curves at high frequencies, therefore having a significantly higher resistance to charge transfer (Figure 3j). Only sulfated GAG‐PEDOT hydrogels presented smaller intercepts and no semicircles. Conductivity values obtained via circuit fitting show that a higher degree of sulfation correlates to higher total conductivity, which accounts for both ionic and electronic contributions to conductivity. Fully re‐protonated HepK‐PEDOT ION hydrogels, having the highest degree of sulfation, showed the highest values of conductivity with 1.65 S m^−1^, followed by the low sulfation CSK‐PEDOT hydrogel ION with a conductivity of 0.56 S m^−1^, and HAK‐PEDOT ION controls with 0.07 S m^−1^ (Figure 3l). Similarly, basal HepK‐PEDOT hydrogel exhibit a conductivity of 0.31 S m^−1^, followed by CSK‐PEDOT with 0.19 S m^−1^ and then HAK‐PEDOT with 0.05 S m^−1^. The magnitude of these values in conductivity are correlated with those observed by four‐point probe measurements (4PP). Using 4PP, differences in ionic conductivity showed the same trends but were found nonsignificant, likely due to less accuracy and the presence of increased resistivity in the edges of the material, which does not occur during EIS measurements.

Overall, it is clear that electronic conductivity in the hydrogel is only originated from the PEDOT domains in the polymers, while ionic conductivity is originated from the ionic species in the glycosaminoglycan backbone. Results strongly suggest that sulfates in the polymer backbone act as acidic self‐doping domains, increasing the conductivity of PEDOT. The doping mechanism would be similar to polystyrene sulfonate (PSS), in which the sulfate groups donate protons to the PEDOT, creating polaron and bipolarons (charge carriers) in the PEDOT chains.^[^ ^25^, ^34^ ^]^ The resulting negatively charged sulfate groups in the backbone polymer stabilize the positive charges on the PEDOT moieties, allowing the formation of a conductive complex with improved electron mobility.^[^ ^25^, ^34^ ^]^ Higher degree of sulfation in the polymer backbone means higher number of protons available for doping the PEDOT side chains, allowing for more polaron formation and higher conductivities. For this reason, the highly sulfated HepK‐PEDOT hydrogels consistently exhibit the highest conductivity levels, while the lower degree of sulfation in CSK‐PEDOT is also correlated to lower conductivity. On the other hand, HAK‐PEDOT is unable to donate protons for electron delocalization and dope PEDOT, hence the conductivity of HAK‐PEDOT hydrogels is the lowest. The further increase in conductivity found in fully re‐protonated polymers (GAG‐PEDOT ION) using ion exchange columns is due to an increase in protons available for doping the PEDOT chains, compared to the basal sulfonated polymer. HAK‐PEDOT does not have sulfonates for re‐protonation and hence treatment with ion exchange columns does not increase conductivity.

### 2.5 Cytocompatibility of Conductive GAG‐PEDOT Oxime Hydrogels

Prior to testing the performance of conductive hydrogels in vitro and in vivo, we evaluated the cytocompatibility of the material. We used cardiomyocytes derived from human induced pluripotent stem cells (hiPSC‐CM), cells responsive to electrical and mechanical stimuli.^[^ ^35^ ^]^ hiPSC‐CM were integrated into the conductive hydrogel as monolayers (2D model) and encapsulated within the hydrogel matrix (3D model). Due to the high charge density of glycosaminoglycans, cardiomyocytes do not adhere properly when seeded directly on top of all preformed hydrogels. Optimal adhesion of a cell monolayer was achieved by first adding fibronectin during hydrogel fabrication and followed by fibronectin coating posthydrogel formation. Performing only one of these steps did not lead to proper monolayer formation (Figure S4, Supporting Information). Cardiomyocyte encapsulation is done by mixing stock solutions of the GAG‐PEDOT, amino‐oxy‐PEG and hiPSC‐CM in cell media supplemented with fibronectin. Of note, full re‐protonation of sulfated glycosaminoglycan‐PEDOT polymers with ion exchange columns caused the death of all seeded or encapsulated cardiomyocytes (Figure S5, Supporting Information), which was due to the high acidity of the polymer (≈pH 2), so GAG‐PEDOT ION polymers were subsequently neutralized with ammonium bicarbonate before hydrogel fabrication and encapsulation.

**Figure 4:**
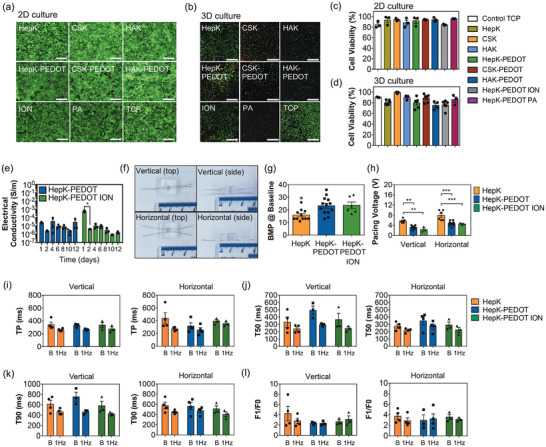
a) Fluorescence microscope images of 2D cultured iPSC‐cardiomyocytes (seeded on the surface of the hydrogels) and b) confocal microscope images of 3D cultured iPSC‐cardiomyocytes (encapsulated within the matrix of the hydrogels) of HepK, CSK, HAK, HepK‐PEDOT, CSK‐PEDOT, HAK‐PEDOT, HepK‐PEDOT ION and HepK‐PEDOT PA hydrogels, as well as tissue culture plastic controls (TCP). Live cells are shown in green (calcein) and dead cells in red (ethidium homodimer). Scale bars represent 200 µm. Quantification of live iPSC‐cardiomyocytes via image analysis of c) fluorescence microscopy images of 2D cultured cells (seeded on the surface of the hydrogels) and d) confocal microscopy images of 3D cultured cells (encapsulated within the matrix of the hydrogels) of HepK, CSK, HAK, HepK‐PEDOT, CSK‐PEDOT, HAK‐PEDOT, HepK‐PEDOT ION and HepK‐PEDOT PA hydrogels. Bars represent the mean (*N* = 3–6) ± SEM. No statistical significance was found, using one‐way ANOVA. e) Stability of electrical conductivity (dry state) over time of basal HepK‐PEDOT and HepK‐PEDOT ION hydrogels incubated in PBS buffer pH 7.4 at 37 °C. Bars represent the mean of three samples (*N* = 3) ± SEM. Statistical significance with (*) *p* < 0.05, using one‐way ANOVA with Tukey post‐tests. f) Electrostimulation devices in a vertical electrode configuration (top) or a horizontal electrode configuration (bottom). The device body is made of PDMS, with a well for hydrogel casting of 5 mm in diameter, while electrodes are made of 0.5 mm platinum wires. g) Spontaneous beating (beats per minute under no electrical stimulation) of iPSC‐cardiomyocytes (GCamPs) seeded on the surface of HepK, HepK‐PEDOT and HepK‐PEDOT ION hydrogels and recorded via fluorescence video‐microscopy in tyrose buffer at 37 °C and 5% CO_2_. Bars represent the mean of pooled samples from hydrogels to be used in the vertical or horizontal electrode configuration (*N* = 6 – 13) ± SEM. Statistical significance with (*) *p* < 0.05, using one‐way ANOVA with Tukey post‐tests. h) Threshold voltage required to pace synchronously iPSC‐cardiomyocytes (GCamPs) seeded on the surface of HepK, HepK‐PEDOT, and HepK‐PEDOT ION hydrogels contained inside the electrostimulation device in either vertical or horizontal electrode configuration, with electrostimulation in tyrose buffer at 37 °C and 5% CO_2_. Bars represent the mean (*N* = 3–6) ± SEM. Statistical significance with (**) *p* < 0.01 and (***) *p* < 0.001, using one‐way ANOVA with Tukey post‐tests. i–l) Calcium traces analyses from fluorescence microscopy video‐recordings of iPSC‐cardiomyocytes (GCamPs) seeded on the surface of HepK, HepK‐PEDOT, and HepK‐PEDOT ION hydrogels contained inside the electrostimulation device in either vertical or horizontal electrode configuration, with electro‐stimulation in tyrose buffer at 37 °C and 5% CO_2_. Traces were obtained using imageJ (NIH, USA) and analyzed with pCLAMP (Molecular Devices, UK) to obtain measurements of time to peak i), time to decay to 50% j) and 90% k), and normalized contracting force l). Bars represent the mean (*N* = 3–6) ± SEM. No statistical significance was found, using two‐way ANOVA.

Cell viability was assessed via LIVE/DEAD staining kit (Thermo Scientific, UK) after 48 h postseeding or encapsulation. Results showed a uniform monolayer of living hiPSC‐CM (green) and minimal presence of dead cells (red) in all hydrogels (**Figure** **4**a). Quantification via image analysis showed that cell viability is highly preserved and similar to tissue culture plastic controls (TCP) with values ranging from 82% to 96% (Figure 4c). Similarly, encapsulated hiPSC‐CM within the hydrogel matrix showed preserved viability, similar to TCP controls, ranging from 76% to 99% (Figure 4b,d). Due to the 3D environment provided by the hydrogel matrix, hiPSC‐CM are mostly rounded, while cells cultured in 2D monolayers showed elongation by 48 h postseeding. Overall, these results confirm that none of the hydrogel polymers are cytotoxic and that any changes in cell viability are only due to standard cell culture conditions.

### 2.6 Electrical Performance of Conductive Hydrogels with hiPSC‐Derived Cardiomyocytes

The stability of the electronic conductivity was assessed over time in conductive hydrogel samples incubated in biological buffers at 37 °C. The basal electronic conductivity of HepK‐PEDOT hydrogels (ranging from 10^−4^ to 10^−6^ S m^−1^) was maintained for the entire length of the study (12 days), as shown in four‐point probe measurements (Figure 4e), which covers the period where hiPSC‐CM were kept in culture and exposed to electrostimulation. On the other hand, the enhanced conductivity of HepK‐PEDOT ION returned to basal levels in less than two days (from ≈10^−3^ to ≈10^−5^ S m^−1^), which is likely a consequence of the sulfate groups exchanging protons with the buffer to restore the acid‐base equilibrium. Regardless, the basal conductivity remains highly stable in biological conditions.

Electrostimulation of hiPSC‐CM was performed using an in house device of fixed dimensions with two electrode configurations, shown in Figure 4f. The horizontal configuration contains both working and counter platinum electrodes in parallel immersed within the hydrogel matrix at a fixed distance. The vertical configuration consists of a working electrode within the hydrogel matrix, while the counter electrode is in contact with the media on the top of the well in the device. For this particular experiment, GCamP‐derived cardiomyocytes were used instead, which is a genetically modified hiPSC line with a fluorescent calcium reporter, hence the action potential of cardiomyocytes can be studied via optical recording of calcium transients directly in the device. Monolayers of hiPSC‐CM were seeded on top of HepK, HepK‐PEDOT, and HepK‐PEDOT ION hydrogels and were maintained in culture for two additional days after cardiomyocytes started spontaneous beating, which in most cases took up to 6 days in total. Cardiomyocytes in monolayers are widely used for electrophysiological studies, with numerous standardized imaging and analysis methods available, while such analyses are more limited and less established for 3D cultures.

In the absence of electrostimulation, the spontaneous beating of cardiomyocytes, or beats per minute (BPM), was found to be higher in conductive HepK‐PEDOT hydrogels (23 ± 2 BMP) and HepK‐PEDOT ION (24 ± 3 BMP), than in HepK hydrogel controls (16 ± 1 BMP), as shown in Figure 4g. Cardiomyocytes in both basal and fully re‐protonated HepK‐PEDOT hydrogels showed similar spontaneous beating. The frequency of the spontaneous beating was not higher than the beating of mature human cardiomyocytes, and hence would not represent a risk for transient post‐transplant ventricular tachycardia if these conductive hydrogels were used as scaffolds for iPSC‐cardiomyocyte implantation.^[^ ^36^ ^]^ The most noticeable finding during electrostimulation was the significantly lower threshold voltage required to achieve pacing of cardiomyocytes in the conductive hydrogels, compared to HepK hydrogel controls in both electrode configurations (Figure 4h). Achieving cardiomyocyte pacing is here referred as cardiomyocytes synchronizing to the frequency of electrostimulation, in which 1 Hz corresponds to 60 beats per minute (BMP). The threshold voltage between basal HepK‐PEDOT and KepK‐PEDOT ION is similar in both electrode configurations, an expected result considering that the increased conductivity of the fully re‐protonated and neutralized HepK‐PEDOT returns quickly to basal levels in presence of biological buffers. In all hydrogels, the threshold voltage required was lower in the vertical electrode configuration, compared to the horizontal configuration, a difference that could be a consequence of the current flow in each of these configurations. In the vertical electrode configuration, the current starts from the working electrode immersed within the hydrogel matrix and must flow through the whole hydrogel and then the cardiomyocyte monolayer before it can reach the counter electrode in contact with the media on the top. In the horizontal electrode configuration, both working and counter electrode are immersed within the hydrogel matrix and hence there is no need for the whole current to flow through the cell monolayer. It is likely that a part of this current goes directly to the counter electrode without reaching cardiomyocytes. It has been noticed that all cardiomyocytes electrostimulated in the vertical configuration have achieved pacing successfully, while a few samples in the horizontal configuration did not properly achieved pacing, in particular those of HepK hydrogels. These control hydrogels also presented a few cardiomyocyte clusters that were not synchronized with the rest of the monolayer and did not respond properly to electrostimulation (Supporting Videos), while no such clusters were present in neither of the conductive HepK‐PEDOT hydrogels.

Overall, results also showed that electrostimulation at 1 Hz decreased the time to peak (TP), time of decay 50% (T50%) and time of decay 90% (T90%) compared to baseline values, regardless of hydrogel type or electrode configuration (Figure 4i–l), while no changes were detected in normalized peak intensity (F1/F0) which is related to the peak calcium of cardiomyocytes. Conductive hydrogels were not found to exert any changes in TP, time of decay and peak calcium compared to HepK hydrogel controls, and hence they do not alter calcium handling within cardiomyocytes. No other differences were observed in calcium handling and action potentials of cardiomyocytes between the vertical and horizontal electrode configuration. The only factor that changed calcium handling in cardiomyocytes was electrostimulation by itself, which is consistent with other electrophysiological studies on electrostimulation of hiPSC‐cardiomyocytes.^[^ ^35^, ^37^ ^]^ On the other hand, the decrease in threshold pacing voltage and increased spontaneous beating of cardiomyocytes in conductive hydrogels showed that they facilitate the electrical propagation of action potentials and provide electrical stability on cardiomyocytes, respectively. Given that the electrical performance of both HepK‐PEDOT and HepK‐PEDOT ION is similar, follow up experiments were performed using the basal HepK‐PEDOT hydrogel.

### 2.7 Injectability and Biodegradation of HepK‐PEDOT Conductive Hydrogels

**Figure 5:**
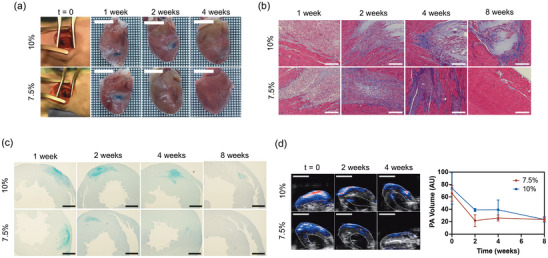
a) Representative images of 10% (w/v) and 7.5% (w/v) HepK‐PEDOT hydrogels injected intramyocardially (*t* = 0), and explanted hearts after 1, 2, and 4 week postimplantation. Scale bars represent 8 mm. b) Masson's Trichrome stained tissue sections of myocardial tissue injected with 10% (w/v) and 7.5% (w/v) HepK‐PEDOT hydrogels after 1, 2, 4, and 8 weeks postimplantation. Scale bars represent 200 µm. c) Alcian Blue stained tissue sections of myocardial tissue injected with 10% (w/v) and 7.5% (w/v) HepK‐PEDOT hydrogels after 1, 2, 4, and 8 weeks postimplantation. Scale bars represent 1500 µm. d) Representative photoacoustic images (PAI) of explanted hearts injected with 10% (w/v) and 7.5% (w/v) HepK‐PEDOT hydrogels after 1, 2, and 4 weeks postimplantation. Images show a slice view from 3D PAI data superimposed on 3D ultrasound, in which the epicardial and endocardial borders are outlined. Scale bars represent 6 mm. The graph shows quantification of hydrogel degradation via photoacoustic signal volume at *t* = 0 and after 2, 4, and 8 weeks postimplantation. Dots represent the mean of five samples (*N* = 4–5) ± SEM.

Oxime crosslinking in the conductive HepK‐PEDOT hydrogels allows hydrogels to be injected after mixing the precursor polymer solutions, HepK‐PEDOT and 8‐arm‐amino‐oxy‐PEG, with or without cells. The gelation kinetics taken via rheological assessments, suggest that the time window for injection, in which the hydrogel mixture is still a liquid, ranges from 5 to 15 minutes, in hydrogels with a concentration of 10% (w/v) to 5% (w/v), respectively (Figure 2h). After this time, the hydrogel gradually increases in viscosity until it becomes a solid, and injection is no longer possible. Gelation should occur once injected into the tissue. Injectability has been tested via intramyocardial injection into the heart of healthy male Sprague Dawley rats, using 10% (w/v) and 7.5% (w/v) HepK‐PEDOT hydrogels (20 µL). Results showed that intramyocardial injection of both concentrations of HepK‐PEDOT is feasible, safe and reproducible. Explanted hearts after injection showed a well‐defined and localized black globule of hydrogel on the myocardium, with no apparent diffusion to adjacent structures or blood vessels (**Figure** **5**a). The time window for injection was appropriate in all cases, giving enough time to mix the precursor polymer solutions, load into the syringe and find the right location and angle for injection. Animals were housed for a period up to 2 months posthydrogel injection and no adverse effects were noticed in any of them. Histological assessment was performed to evaluate tissue remodelling via Masson's Trichrome staining, while hydrogel evolution was followed via Alcian Blue staining, which is specific for glycosaminoglycans.

At 7 days postimplantation, histological stained tissue sections showed that the hydrogel has been injected within the myocardium, where the surrounding tissue structures are preserved, with no signs of tissue necrosis, swelling or exacerbated inflammation (Figure 5b). Interestingly, significant cell infiltration was observed within the hydrogel matrix, in all time‐points where hydrogels were present, indicating tissue remodeling. Over time, gradual degradation of the conductive hydrogels was observed, until complete disappearance, with a degradation rate dependent of hydrogel concentration. In 10% (w/v) HepK‐PEDOT hydrogels, the amount of hydrogel present at 14 days post injection was shown to decrease compared to 7 days (Figure 5c). Only a few discrete zones with hydrogels were observed at 4 weeks, while no hydrogel was found in most samples by 8 weeks postimplantation. In 7.5% (w/v) HepK‐PEDOT hydrogels, degradation was found to be faster. At 14 days, a reduced presence of hydrogel was observed, in a similar degree to that of 10% (w/v) hydrogels after 4 weeks. At 4 weeks, most tissue samples did not present hydrogels. Encapsulation around implanted hydrogels was not observed in any of the histological samples stained with Masson's Trichrome, which is likely a consequence of the biodegradation (Figure 5b). Encapsulation of implanted materials is common in nondegradable materials and materials which degradation does not match with the remodeling rate of the surrounding tissue, which is closely linked with a chronic inflammatory response.^[^ ^38^ ^]^

Given the dark pigmentation of the crosslinked HepK‐PEDOT hydrogels, we investigated whether it could generate photoacoustic signals that may allow tracking of its location and degradation. Spectral scanning of crosslinked HepK‐PEDOT hydrogel phantoms identified maximal signal generation at 800 nm. Although PAI can be performed in vivo in mouse hearts,^[^ ^39^ ^]^ it is more challenging in rats owing to greater signal attenuation from the thicker chest wall. Hence, hearts were explanted at different time points after injection and 3D PAI was performed on the excised organs. Images and quantification of the volume of photoacoustic signal (PAI volume) showed a pronounced signal decay within the first 2 weeks postimplantation, followed by a slower clearance of the signal (Figure 5d), which agrees with the degradation of the hydrogels. Also, the 10% (w/v) hydrogels seem to remain in the heart tissue for longer than the 7.5% (w/v) hydrogels. Interestingly, while histological and visual examination of the hearts show little to no presence of hydrogels after 4 weeks postimplantation, we still observed some PAI signal within the heart tissue. It is likely that this remnant signal is given by the degraded conductive polymer, which is cleared out the tissue at a slower pace. Afterall, the PA signal originates from the conductive polymer rather than the hydrogel itself. No bioaccumulation was observed, indeed both the hydrogel under histology and the PAI signal disappear gradually over time, regardless of the different clearing rates. Therefore, HepK‐PEDOT conductive hydrogels are biodegradable, and the rate of degradation can be tuned with polymer concentration, as lower concentrations degrade faster. The mechanisms of degradation in the conductive hydrogels likely involve the catalytic activity of the infiltrated cells and secretion of heparinases, but the specific mechanisms remain to be elucidated. However, given that heparinases break heparin in smaller fragments, it is likely that the PEDOT moieties in the copolymer are excised as smaller fragments of heparin oligosaccharides bound to PEDOT, which would be water‐soluble and cleared out without metabolization.

Degradation of HepK‐PEDOT hydrogels was assessed in vitro using heparinases I, II, and III at 37 °C, however, their enzymatic activity was not sufficient to degrade the hydrogel in a measurable manner. It has been claimed that these enzymes in fact do not quantitatively digest the whole molecule and possess short half‐life (12–18 min),^[^ ^40^ ^]^ and for this reason, standard experiments only monitor degradation of a few micrograms of heparin for a short period of time (minutes). In the conductive hydrogels, heparin is at the milligram scale and the enzymes need to be active for longer time, which in this case is not feasible, even with frequent changes of fresh enzyme. Alternatively, poor in vitro degradation may be a consequence of steric hindrance provided by PEDOT side‐chains, not enabling the enzymes to reach the target sugar sequences within the heparin backbone. It is likely that poor in vitro degradation is a consequence of both of these problems. Interestingly, the conductive hydrogels were found to redissolve in water within a period of 24 h in vitro, while hydrogels are indefinitely stable in physiological buffers, being unchanged for at least two months. This process in water starts with progressive swelling, until the hydrogel does not hold its structure and breaks down in solution (Figure S6, Supporting Information). Another interesting question arising from these biodegradation experiments is related to the electrical stability of conductive hydrogels postimplantation. Compared to in vitro conditions, the microbiological environment of tissues contains immune cells, ionic species, enzymes, and other proteins; suggesting that the electrical properties of conductive hydrogels may decline faster. Future studies using electrostimulation of conductive hydrogels in vivo will reveal the impact of biodegradation on the electrical properties of both conductive hydrogels and cardiomyocytes, and the length in which conductive hydrogels would meet the required electromechanical performance in a specific clinical application. Therefore, our findings in animal studies demonstrate that conductive hydrogels can be injected, gel in situ, biodegrade and not elicit any undesirable effects, suggesting biocompatibility. However, further studies should be performed to assess the host response more exhaustively, which includes the immune response and cardiac function in vivo.

## 3 Conclusion 

PEDOT side‐chain growth polymerization is a suitable technique to obtain conductive brush copolymers of sulfated polysaccharides and PEDOT, as well as in situ on preformed hydrogels. PEDOT side‐chain growth occurs on the grafted EDOT‐NH_2_ monomer in the glycosaminoglycan backbone chain, via radical initiated polymerization in aqueous conditions. Similarly, the side‐chain growth of PEDOT can be performed in preformed hydrogels (in situ), after EDOT‐NH_2_ grafting. The rate and extent of PEDOT polymerization are accelerated with an increasing sulfation degree in the glycosaminoglycan backbone chain, while completion is not reached in the absence of sulfation in the polymer. Conductive hydrogels with superior mechanical and electronic properties are obtained when grafting of both EDOT‐NH_2_ monomer and crosslinker are performed together in one single step, rather than sequentially. As expected from oxime‐based hydrogels, the stiffness and gelation kinetics of the conductive hydrogels were shown to be tunable with both concentration and pH, with lower concentration and pH delaying the onset of gelation. Moreover, conductive hydrogels were shown to possess adhesive properties. Due to the reversibility of oxime‐crosslinking, conductive hydrogels also exhibited self‐healing properties.

Conductive glycosaminoglycan‐PEDOT hydrogels exhibited both ionic and electronic conductivity. The presence of sulfate groups in the glycosaminoglycan backbone chains were shown to act as doping moieties, with a higher sulfation degree increasing the conductivity of hydrogels. Also, full re‐protonation of sulfate groups in the polymer, via ion exchange column treatment, increased further the conductivity of GAG‐PEDOT hydrogels. However, this increased conductivity resulted in high acidity and cytotoxicity, which was solved by neutralization. On the other hand, basal conductive hydrogels and nonconductive controls were shown to preserve cell viability of iPSC‐derived cardiomyocytes both seeded as monolayers and encapsulated within the hydrogel matrix. Regardless treatment, all conductive hydrogels facilitated the propagation of action potentials in iPSC‐cardiomyocytes in the absence and the presence of electrostimulation, as evidenced by the increased spontaneous activity of cardiomyocytes and the lower pacing threshold voltage observed in the conductive hydrogels. Importantly, the conductivity was shown to be highly stable in biological conditions during the whole course of the study. Moreover, electrostimulation was the only factor affecting calcium handling in cardiomyocytes, while conductive hydrogels did not exert any significant effects.

Implantation of the conductive hydrogels via intramyocardial injection was successfully and reproducibly performed, owing to the mixed precursor materials being liquid during injection, followed by gelation within the heart tissue. The conductive hydrogels are biocompatible, as they preserve the structure of the myocardium with no signs of encapsulation, injury or implant‐related complications in any of the animals in the study. They were shown to be biodegradable, presenting significant cell infiltration at early time points and progressively degrading until the tissue remodeling process and hydrogel disappearance is completed. The rate of hydrogel degradation was found to be correlated with the total mass concentration. Overall, the results of this study demonstrate that aiming for high conductivity values in conjugated polymers is not as important as obtaining values that are compliant with ranges in conductivity found in native tissues, and hence we suggest focusing on the development of conductive materials that aim to recapitulate both the architecture and the functionality of native tissues.

## 4 Experimental Section

### Materials

Heparin, chondroitin sulfate A, *N*‐(3‐dimethylaminopropyl)‐*N*′‐ethylcarbodiimide hydrochloride (EDC), *N*‐Hydroxysuccinimide (NHS), *N*‐hydroxyphthalimide, ammonium persulfate (APS), oxalyl chloride, *N*‐bromosuccinimide (NBS), ethylenediamine, di‐tert‐butyl decarbonate, triphenylphosphine, diisopropyl azodicarboxylate (DIAD), hydrazine monohydrate, triethylamine and trifluoroacetic acid (TFA) were purchased from Sigma‐Aldrich. Hyaluronic acid was purchased from Lifecore Biomedical. EDOT, aminoacetone and palladium (II) acetate were purchased from Fluorochem. Sylgard 184 elastomer kit, dioxane, DCM, hexane, ethyl acetate, HPLC‐grade water, K_2_CO_3_, MgSO_4_, deuterium oxide and deuterochloroform were purchased from VWR. 8‐arm PEG‐OH (40 KDa) was obtained from Creative PEG Works. Aminoethyl methacrylate (AEMA) was purchased from Polysciences, Inc. Snakeskin dialysis membranes, 3.5 KDa were purchased from ThermoFisher Scientific. PD‐10 desalting columns were purchased from General Electric.

### EDOT‐NH_2_ Monomer Synthesis

To introduce amino groups in an EDOT monomer, a Boc‐protected ethylenediamine was synthesized. A solution containing 45 mmol (0.5 eq) of di‐*tert*‐butyl dicarbonate (Boc‐anhydride) in 200 mL of DCM was added to a 90 mmol (1 eq) solution of ethylediamine in 50 mL of DCM during the course of 5 h using a dropping funnel. After stirring overnight, the pure Boc‐protected ethylene diamine was obtained by washing twice with 2 m K_2_CO_3_ in a separation funnel, then dried with MgSO_4_, filtered, rotavaped and left under vacuum overnight.

To synthesize the EDOT‐NH‐Boc intermediate, 10 mmol (1 eq) of EDOT were dissolved in 30 mL of dioxane and 10 mmol (1 eq) of oxalyl chloride were added dropwise. The mixture was heated to 100 °C under stirring for 1 h and then allowed to cool down to room temperature (RT). Once ready, 15 mmol (1.5 eq) of Boc‐protected ethylenediamine and 50 mmol (5 eq) of triethylamine were added to the mixture and stirred during 4 h. The mixture was diluted in 150 mL of DCM, washed two times with 100 mL of water in a separation funnel, and the organic phase dried with MgSO_4_, filtered and rotavaped to concentrate. The crude was then purified using flash column chromatography using ethyl acetate:hexane 65:35 as mobile phase. EDOT‐NH‐Boc powder product was obtained by rotary evaporation and vacuum drying.

To obtain the final EDOT‐NH_2_ monomer, Boc deprotection was done by adding dropwise 20 eq of trifluoroacetic acid (TFA) to a solution containing 1 eq of EDOT‐NH‐Boc at 10 mg mL^−1^ in DCM, stirring for 1 h at RT. The mixture was then concentrated in a rotary evaporator and re‐diluted in DCM two times, then rotavaped until dry and kept in vacuum for 24 h. The dry product was then dissolved in HPLC‐grade water, passed through a PD‐10 desalting column and freeze dried. Before use, the pure EDOT‐NH_2_ powder was redissolved in HPLC‐grade water at 100 mg mL^−1^ and adjusted to pH 6.5.

### Glycosaminoglycan‐PEDOT Synthesis

Grafting of EDOT‐NH_2_ monomer to the glycosaminoglycan (GAG) was done via EDC/NHS coupling in an equivalent GAG:EDC:NHS:monomer ratio of 1:5:5:2, with a total GAG concentration of 10 mg mL^−1^ in HPLC‐grade water. In a typical reaction, 100 mg of GAG were dissolved in 2.5 mL of HPLC‐grade water. EDC (170 mg in 2.5 mL of water) and NHS (106 mg in 2.5 mL of water) were added to the GAG solution and stirred for 15 min at room temperature (RT). EDOT‐NH_2_ (90 mg in 2.5 mL of water) was added dropwise to the mixture and left stirring overnight at RT. The GAG‐EDOT product was purified through dialysis using a 3.5 KDa membrane against milliQ‐water for 4 days, then passed through a PD‐10 desalting column and freeze dried. Degree of functionalization (DoF) was determined by dissolving the polymer at a concentration of 1 mg mL^−1^ in H_2_O, measuring absorbance at 320 nm in a UV/Vis reader, and extrapolating to a standard curve of the EDOT‐NH_2_.

The GAG‐PEDOT polymer was obtained via side‐chain growth polymerization using the grafted EDOT as seeding point and a radical initiator. In a typical reaction, 100 mg of GAG‐EDOT were dissolved at a concentration of 5 mg mL^−1^ in HPLC‐grade water. Under vigorous stirring, 320 mg of ammonium persulfate (APS), dissolved in a minimal amount of water, were added to the solution followed by 36 µL of EDOT. The reaction was put under N_2_ atmosphere and left stirring for 24 h at room temperature. The solution was passed through a 0.2 µm filter to remove the nonsoluble free PEDOT impurities. The GAG‐PEDOT product (Hep‐PEDOT, CS‐PEDOT or HA‐PEDOT) was further purified through dialysis using a 3.5 KDa membrane against milliQ‐water for 4 days, then passed through a PD‐10 desalting column and freeze dried.

Enhanced doping of heparin‐PEDOT was done by protonating all sulphate groups in heparin, passing a 20 mg mL^−1^ solution of the polymer in water through a Dowex 50WX8 (hydrogen form, 200–400 mesh) ionic exchange column, followed by freeze drying. Alternatively, GAG‐PEDOT hydrogels were immersed for 30 min in either 1 M of phytic acid, methane sulfonic acid or HCl. During experiments with iPSC‐derived cardiomyocytes, the low pH (≈2) of the fully protonated GAG‐PEDOT polymers was neutralized using ammonium bicarbonate, followed by freeze drying. In case of casted hydrogels, the acid was removed by extensive washes in water.

### Glycosaminoglycan‐Ketone Synthesis

Grafting of the aminoacetone to the glycosaminoglycan (GAG) was done via EDC/NHS coupling in an equivalent GAG:EDC:NHS:aminoacetone ratio of 1:5:5:2, with a total GAG concentration of 10 mg mL^−1^ in HPLC‐grade water. In a typical reaction, 100 mg of GAG were dissolved in 2.5 mL of HPLC‐grade water. EDC (170 mg in 2.5 mL of water) and NHS (106 mg in 2.5 mL of water) were added to the GAG solution and stirred for 15 min at room temperature (RT). Aminoacetone (40 mg in 2.5 mL of water) was added dropwise to the mixture and left stirring overnight at RT. The GAG‐ketone product (HepK, CSK or HAK) was purified through dialysis using a 3.5 KDa membrane against milliQ‐water for 4 days, then passed through a PD‐10 desalting column and freeze dried.

### Glycosaminoglycan‐Ketone‐PEDOT Synthesis

The synthesis of GAG‐ketone‐PEDOT has been performed either in sequence or combination as follows:

Sequence A: 1) EDOT grafting, 2) GAG‐PEDOT side‐chain polymerization, and 3) Ketone grafting

Sequence B: 1) Ketone grafting, 2) EDOT grafting, and 3) GAG‐PEDOT side‐chain polymerization

Combination: 1) Dual EDOT/ketone grafting and 2) GAG‐ketone‐PEDOT side‐chain polymerization

Dual grafting of EDOT‐NH_2_ monomer and aminoacetone (crosslinker) to the glycosaminoglycan (GAG) was done via EDC/NHS coupling in an equivalent GAG:EDC:NHS:monomer:aminoacetone ratio of 1:10:10:2:2, with a total GAG concentration of 10 mg mL^−1^ in HPLC‐grade water. In a typical reaction, 100 mg of GAG were dissolved in 2.5 mL of HPLC‐grade water. EDC (340 mg in 2.5 mL of water) and NHS (212 mg in 2.5 mL of water) were added to the GAG solution and stirred for 15 min at room temperature (RT). A solution containing both EDOT‐NH_2_ (90 mg) and amino acetone (40 mg) in 2.5 mL of water was added dropwise to the mixture and left stirring overnight at RT. The GAG‐ketone‐EDOT product was purified through dialysis using a 3.5 kDa membrane against milliQ‐water for 4 days, then passed through a PD‐10 desalting column and freeze dried.

The GAG‐ketone‐PEDOT polymer (HepK‐PEDOT, CSK‐PEDOT or HAK‐PEDOT) was obtained via side‐chain growth polymerization using the grafted EDOT as seeding point and a radical initiator. In a typical reaction, 100 mg of GAG‐ketone‐EDOT were dissolved at a concentration of 5 mg mL^−1^ in HPLC‐grade water. Under vigorous stirring, 320 mg of ammonium persulfate (APS), dissolved in minimal amount of water, were added to the solution followed by 36 µL of EDOT. The reaction was put under N_2_ atmosphere and left stirring for 24 h at room temperature. The solution was passed through a 0.2 µm filter to remove the nonsoluble free PEDOT impurities. The GAG‐ketone‐PEDOT product was further purified through dialysis using a 3.5 KDa membrane against milliQ‐water for 4 days, then passed through a PD‐10 desalting column and freeze dried. Enhanced doping was done as previously described.

### PEDOT Side Chain Growth Kinetics

To optimize the polymerization and study the growth kinetics of the PEDOT side‐chains from heparin, different APS to monomer ratios were used: 16:1, 8:1, 4:1, 1:1, 1:4, 1:8, 1:16, and a no‐APS control. In all conditions, the amount of GAG‐PEDOT (at 5 mg mL^−1^ in H_2_O) and EDOT were fixed, only changing the amount of APS for each specific ratio. All reactions were vigorously stirred under N_2_ atmosphere. Aliquots were taken every hour for 8 h, then at 24 h. Aliquots were diluted twice in H_2_O and spectra from 280 to 1000 nm were acquired for further data analysis, using a SpectraMax M5 UV/Vis plate reader (Molecular Devices, UK).

### 8‐Arm PEG‐O‐NH_2_ Synthesis

In a flame dried round flask, 1 g (1 eq) of 8‐arm PEG‐OH (40 KDa) was dissolved in 40 mL of anhydrous DCM, then 636 mg of *N*‐hydroxyphthalimide were added. Triphenylphosphine (1 g) was added and the flask was cooled down to 4 °C. A precooled solution containing 766 µL diisopropyl azodicarboxylate (DIAD) in 5 mL of anhydrous DCM was added slowly dropwise to the mixture under vigorous stirring, and left stirring overnight. The intermediate 8‐arm PEG‐phthalimide was obtained by precipitating and redissolving three times the polymer in cold diethyl ether. The precipitated product was then dried overnight under vacuum.

To obtain the final 8‐arm PEG‐O‐NH_2_, 1 g of 8‐arm PEG‐phthalimide was dissolved in 20 mL of acetonitrile. Under stirring, 368 µL of hydrazine monohydrate were added to the solution and stirred for 2 h. The mixture was left to evaporate under N_2_ stream. The crude powder was redissolved in 10 mL of DCM and filtered through a Celite 545 plug. The crude product was then dried via rotary evaporation, redissolved in 10 mL of DCM, precipitated in cold diethyl ether and dyed overnight under vacuum to obtain a pure powder.

### Oxime Hydrogel Fabrication

The GAGK‐PEDOT/GAGK polymer was mixed with 8‐arm PEG‐O‐NH_2_ in equimolar ratios between ketones and amino‐oxy groups. For example, a 30 µL of 10% (w/v) (total mass) HepK‐PEDOT conductive hydrogel, was made by mixing 1.3 mg of HepK‐PEDOT in 15 µL of H_2_O/PBS, with 1.7 mg of 8‐arm PEG‐O‐NH_2_ in 15 µL of H_2_O/PBS. Oxyme crosslink formation and hydrogel gelation occurs in ≈10 min. Hydrogels were made at different concentrations and PBS was used at different pHs. To encapsulate cells, each polymer was dissolved in 12.5 µL of media and mixed with 5 µL of the cell suspension.

### Mechanical Testing

Hydrogel gelation kinetics was performed in an Anton Paar MCR 302 Rheometer, mixing and immediately placing 30 µL of the liquid hydrogel components into the instrument, using an 8 mm plate and a gap of 0.5 mm. Storage (G') and loss (G″) modulus data from three different batches was acquired at 0.5 Hz, 1% strain and 25 °C, until reaching plateau. Hydrogel stiffness was determined from samples from three batches at 0.5 Hz, 1% strain and 25 °C, parameters located within the linear viscoelastic region, corroborated by performing a frequency sweep from 0.01 Hz to 10 Hz at 1% strain, and a strain sweep from 0.0001% to 10% at 0.5 Hz.

### Self‐Healing Assessments

To assess self‐healing capacity of oxime hydrogels, one KepK‐PEDOT hydrogel and one HepK hydrogel were fabricated in cylindrical 8 mm molds at a concentration of 10% (w/v) in PBS and volume of 40 µL. Both hydrogels were cut in halves and one half of HepK‐PEDOT hydrogel was put in contact with a half of HepK hydrogel, pressing both of them with forceps for 1 min. Hydrogels were left in a sealed tube and observed during 6 h. Rheological assessment of self‐healing was done in an Anton Paar MCR 302 Rheometer, mixing and immediately placing 30 µL of the liquid hydrogel components into the instrument, using a 8 mm plate and a gap of 0.5 mm. Before running the test, completed hydrogel gelation was corroborated by reaching a plateau in both storage (G') and loss (G″″) modulus at 0.5% strain, 0.5 Hz and 25 °C. Then high strain forces to break the hydrogel were done at 500% strain, 0.5 Hz and 25 °C for 1 min, followed by a recovery period of 1 minute at 0.5% strain, 0.5 Hz and 25 °C, to allow hydrogels to recover their initial mechanical properties. Hydrogel rupture was confirmed by the inversion of both G' and G″″ curves. This cycle was repeated 6 times.

### Adhesion Testing

Conductive hydrogels at concentration of 10% (w/v) and volume of 80 µL were casted between two glass slides, with hydrogels sandwiched in an area of 25 mm × 25 mm. After gelation, each slide free‐end was clamped between the top and bottom holder of a ElectroForce 3200 Test System (TA Instruments, UK) to perform an uniaxial pull‐out test. Slices were pulled apart the hydrogel separated from one of the slides. Force‐time curves were recorded and transformed to force–distance curves using WinTest Advance Control Software V8.0. The adhesive strength (kPa) of conductive hydrogels was estimated from the ratio of maximum recorded force to the initial hydrogel‐glass contact area.

### Electrical Characterization

Hydrogels (10% (w/v), 30 µL in H_2_O) were casted into circular molds of 8 mm in diameter and 500 µm thickness. Once fully gelled, conductivity of hydrated hydrogels was measured on a 4‐point probe (Ossila, UK), which is the sum of both electric and ionic conductivity. Hydrogels were then freeze dried and conductivity was measured again to obtain electrical conductivity only. Ionic conductivity was calculated as a difference between the total and electric conductivity.

Electrochemical Impedance Spectroscopy (EIS) was performed on 40 µL, 10% (w/v) hydrogels in H_2_O, mounted between two glassy carbon electrodes of 6 mm diameter, forming a gap of 3 mm. Impedance was measured over a range of 10^−2^ Hz to 10^6^ Hz, at 50 mV, 20 points per decade.

Cyclic voltammetry was performed on 10% (w/v) hydrogels (80 µL, in PBS) on the top of a glassy carbon electrode (1 mm in diameter) within an electrochemical cell containing PBS as electrolyte solution and a AgCl_3_ reference electrode. Measurements were done from −0.2 V to +0.8 V, 0.01 V step size and a range of frequencies from 10 to 200 mV s^−1^.

### iPSC‐Cardiomyocyte Culture and Differentiation

Two different iPSC lines were used in this study. IMR90 cells (WiCell, USA) were used for cytocompatibility assessment, while GCamP‐WTC‐11 cells (J. David Gladstone Institutes, USA), expressing a fluorescently green calcium reporter, were used for electrostimulation studies. Both cell lines were cultured in Essential 8 (E8) medium (Gibco, UK) at 37 °C and 5% CO_2_. Detachment of cells was performed with 0.5 × 10^−3^ m EDTA in PBS for 5 min at room temperature. EDTA was removed and E8 media with thiazovivin (10 × 10^−3^ m) was added to remove cells by pipetting. Cells were seeded in Matrigel‐coated plates at a 1:20 split ratio. Once reaching 85% confluency, differentiation was started by adding RPMI 1640 + B27 without insulin + 6 × 10^−6^ m CHIR99021 for two days. Media was changed to RPMI 1640 + B27 without insulin for 1 day. Media was then changed to RPMI 1640 + B27 without insulin + 2.5 × 10^−6^ m Wnt‐C59 for two days. Media was changed again to RPMI 1640 + B27 without insulin for two days. Media was then changed to RPMI 1640 + B27 and changed every two days, until cell beating is observed. Metabolic selection was done by feeding cells with RPMI 1640 without glucose + B27 for four days, with changes every two days. Resulting cardiomyocytes were kept in RPMI 1640 + B27 and changed media every two days. iPSC‐derived cardiomyocytes detachment was done with collagenase II (200 U mL^−1^), 1 mM HEPES and 10 × 10^−3^ m thiazovivin for 3 h, following by gentle pipetting. An equivalent volume of DNAse I (1:500) in RPMI 1640 media was added to inactivate collagenase II. Cells were centrifuged at 100 g for 15 min and resuspended in RPMI 1640 + B27 media to desired cell concentration.

### Cell Viability

iPSC‐cardiomyocytes (IMR90) were resuspended in RPMI 1640 + B27 media, 20% (v/v) FBS, 10 mM thiazovivin and 1% Penicilin/Streptomycin (PS) before seeding or encapsulation. For cells seeded as monolayers, conductive hydrogels and controls were fabricated the previous day using PBS + 5% (w/v) fibronectin as solution to dissolve all polymers. Once hydrogels were formed in a cylindrical 8 mm mold, 40 µL of 5% (w/v) fibronectin in PBS were added on top of hydrogels and incubated overnight at 4 °C. Next day, hydrogels were washed three times in PBS. A drop of 50 µL containing 3 × 10^4^ iPSC‐cardiomyocytes was seeded on top of the hydrogels and incubated for 3 h at 37 °C and 5% (v/v) CO_2_ to allow cell adhesion, then the whole 24‐well was filled with RPMI 1640 + B27 media, 20% (v/v) FBS, 10 mM thiazovivin and 1% (v/v) Penicilin/Streptomycin (PS). Next day, cells media was changed to RPMI 1640 + B27 and incubated for a total of 48 h at 37 °C and 5% (v/v) CO_2_. After 48 h, the viability of iPSC‐cardiomyocytes was assessed using LIVE/DEAD staining kit (Thermo Fisher, UK), following kit instructions. Assessment was performed via fluorescence microscopy (Olympus BX51 microscope. Olympus, UK) and image analysis quantification via ImageJ (NIH, USA).

In case of iPSC‐cardiomyocyte encapsulation, all glycosaminoglycan‐PEDOT and 8‐arm amino‐oxy‐PEG stock solutions were dissolved in RPMI 1640 + B27, 20% (v/v) FBS, 10 × 10^−3^ m thiazovivin, 1% (v/v) PS, 1 × 10^−3^ m HEPES and 5% (w/v) fibronectin. Polymer solutions were mixed with 3 × 10^4^ iPSC‐cardiomyocytes in a total hydrogel volume of 40 µL. Once hydrogels were formed, the well was filled with RPMI 1640 + B27 media, 20% (v/v) FBS, 10 × 10^−3^ m thiazovivin and 1% (v/v) Penicilin/Streptomycin (PS). Cells were incubated overnight and then changed to RPMI 1640 + B27 and incubated for a total of 48 h at 37 °C and 5% (v/v) CO_2_. After 48 h, the viability of iPSC‐cardiomyocytes was assessed using live/dead staining kit (Thermo Fisher, UK), following kit instructions. Assessment was performed via confocal microscopy (Leica TCS SP5, Leica Microsystems, UK) and image analysis quantification via ImageJ (NIH, USA).

### Electrostimulation Device Fabrication

Molds containing a cylinder of 5 mm in diameter were fabricated using a masked soft photolithography (MSLA) 3D Printer (Prusa SL1S, Czech Republic). Sylgard 184 was mixed in a ratio of 1:10 (initiator: resin), degassed and poured in the molds. Curing was done overnight in an oven at 60 °C. The resulting PDMS device and a glass slide were plasma treated at 100 W, 2 mBar O_2_ gas pressure for 1 min, then bonded for 2 h in an oven at 60 °C. Two platinum wires (0.5 mm diameter) were inserted in the base of the well within a distance of 3 mm of each other (horizontal configuration) or vertically aligned from each other – one in the base of the device and the other 3 mm above the top surface of the hydrogel. Images of the electrostimulation devices are shown in Figure 4f. The finished devices were sterilized by dipping in pure isopropanol and dried in sterile conditions.

### Cardiomyocyte Electrostimulation

Conductive hydrogels and controls were fabricated the previous day using PBS + 5% (w/v) fibronectin as solution to dissolve all polymers. 40 µL hydrogels were formed in the 5 mm well within the electrostimulation device and waited until gelation. A 40 µL drop of 5% (w/v) fibronectin in PBS was added on top of the hydrogels and incubated overnight at 4 °C. Next day, hydrogels were washed three times in PBS. In parallel, iPSC‐cardiomyocytes (GCamPs) were resuspended in RPMI 1640 + B27 media, 20% (v/v) FBS, 10 × 10^−3^ m thiazovivin and 1% (v/v) Penicilin/Streptomycin (PS) before seeding. A drop of 50 µL containing 3 × 10^4^ iPSC‐cardiomyocytes was seeded on top of the hydrogels and incubated for 3 h at 37 °C and 5% (v/v) CO_2_ to allow cell adhesion, then the whole well inside the device was filled with RPMI 1640 + B27 media, 20% (v/v) FBS, 10 mM thiazovivin and 1% (v/v) Penicilin/Streptomycin (PS). Next day, cells media was changed to RPMI 1640 + B27 and incubated at 37 °C and 5% (v/v) CO_2_ for four days. This time allowed iPSC‐cardiomyocytes recover their original morphology and restart beating (usually within two days), and extra two days to fully adjust to their new environment before electrostimulation. The media in the well of the electrostimulation device was changed to 1X Tyrose buffer at 37 °C. The working and counter electrodes in the electrostimulation device were connected to a MyoPacer stimulator (Ionoptix, Ireland), within an incubating chamber of a fluorescence widefield microscope (Zeiss Axio Observer Z1) and kept at 37 °C and 5% (v/v) CO_2_ for the whole duration of the experiments. Cardiomyocytes in the hydrogels were stimulated at frequency of 1 Hz, pulse duration of 20 ms and increasing voltage steps of 0.5 V, until reaching cardiomyocyte pacing. Calcium fluorescent traces were recorded via video microscopy for 60 sec. Data from recordings was extracted with ImageJ (NIH, USA) and analyzed with pCLAMP software V11 (Molecular Devices, USA).

### Hydrogel Injectability, Biocompatibility, and Degradation

Conductive hydrogels were injected intramyocardially and assessed in Sprague‐Dawley male rats (250–350 g, Charles River Laboratories), with a minimum acclimatization of one week before the procedure. All animal procedures were performed under license by the UK Home Office, in agreement with the United Kingdom Animals (Scientific Procedures) Act 1986 and guidelines established by the European Directive on the protection of animals used for scientific purposes (2010/63/EU). Animals were anesthetized with 5% (v/v) isoflurane (Zoatis, UK), confirmed by observing the loss of standing and pedal reflexes. The fur from the thorax was removed using clippers, the skin disinfected with betadine, and eyes hydrated with Lacri‐lube (Allergan, UK). Buprenorphine at 0.05 mg k^−1^g (Vetergesic, Ceva UK) was diluted in saline and injected subcutaneously to provide analgesia and fluids. Rats were cannulated and put under mechanical ventilation (Harvard Apparatus, UK), using parameters adjusted to maintain an oxygen saturation of 97–99% and a heart rate of 300–400 bpm. The whole surgical procedure was carried out using 2% isoflurane and body temperature maintained with a heating mat. A horizontal incision of the skin, followed by dissociation of the intercostal muscles and expansion of the space between the ribs of the thorax were performed to expose the left ventricle of the heart. The pericardium was ruptured to facilitate visibility of the myocardium. Both HepK‐PEDOT and 8‐arm‐PEG‐O‐NH_2_, previously and separately dissolved in 1X PBS, were mixed and loaded into a 21G Halmiton Syringe (Hamilton, UK). A shallow injection of 20 µL of 7.5% (w/v) or 10% (w/v) conductive hydrogel was done below the surface of the myocardium, resulting in a confined circular area of hydrogel. After gelation (10 minutes), the muscles and skin were closed with 4/0 resorbable sutures (PDSII, Ethicon, Belgium). Isoflurane anesthesia was decreased to 1% during skin suturing, then turned off at the end of the surgery to allow the animal to recover, while extubated and put in a heated oxygen chamber until full recovery. Pain and distress were monitored regularly, while Buprenorphine (0.05 mg k^−1^g, Vetergesic, Ceva, UK) was administered for one day to provide analgesia.

After 1, 2, 4 or 8 weeks postprocedure, the rats were euthanized and the chest was opened to explant the heart. Explanted hearts were put into a heparinized solution and fixed with 4% PFA overnight at room temperature, then kept in 70% ethanol at 4 °C before photoacoustic imaging (PAI). PAI images were acquired using a high‐frequency ultrasound and nano‐second pulsed laser system (Vevo 3100, VevoLAZR, FujiFilm VisualSonics Inc., Toronto). Excised hearts were immersed in water and excitation wavelength was swept from 680 to 940 nm. Maximal signal from the conductive polymers was generated at 800nm. 3D volumetric images covering the entire heart were then acquired at 800 nm with a constant PA gain of 40. PAI volume was determined using FujiFilm VisualSonics’ VevoLab software. All 3D images were thresholded to 45% and the volume of PAI signal above this threshold was calculated across the whole heart.

### Statistical Analysis

Comparisons of experimental groups were performed using two‐tailed *t*‐test, one‐way analysis of variance (ANOVA) or two‐way ANOVA, using at least *p* < 0.05 as statistical significance criteria, followed by post hoc tests to perform multiple comparisons. Sample size (*n*) is shown individually for each experiment in the figure captions. Statistical analyses were performed on GraphPad Prism V8 (California, USA). Shapiro–Wilk tests were used to corroborate normality of data, using SPSS V28 (IBM, USA).

## Conflict of Interest

M.M.S. invested in consults for (or is on scientific advisory boards or boards of directors) and conducts sponsored research funded by companies related to the biomaterials field. The rest of the authors declare no conflict of interests.

## Ethical Approval Statement

All animal procedures were performed under licenses PEE7C76CD and PP1692884 by the UK Home Office, in agreement with the United Kingdom Animals (Scientific Procedures) Act 1986 and guidelines established by the European Directive on the protection of animals used for scientific purposes (2010/63/EU). The GCaMP‐iPSCs, from WTC‐11 line was a kind gift from Professor Bruce Conklin, The J. David Gladstone Institutes, USA. This line was generated from a healthy male donor who signed a consent form. The protocol was approved by the UCSF Committee on Human Research, San Francisco, USA (study number 10–02521, “Induced Pluripotent Stem Cells for Genetic Research”). IMR‐90 iPSC line (catalog #IMR90) was acquired commercially from WiCell (USA), provided by Prof. James Thomson at University of Wisconsin, and licensed for academic use.

## Supporting information



Supporting Information

Description of Supplementary Videos

Supplemental Movie 1

Supplemental Movie 2

Supplemental Movie 3

Supplemental Movie 4

Supplemental Movie 5

Supplemental Movie 6

Supplemental Movie 7

Supplemental Movie 8

Supplemental Movie 9

Supplemental Movie 10

Supplemental Movie 11

Supplemental Movie 12

## Data Availability

For the purpose of open access, the author has applied a Creative Commons Attribution (CC:BY) licence to any Author Accepted Manuscript version arising. Raw research data is available upon request from the corresponding author.
