# Supplementary material for: Self‐Doped and Biodegradable Glycosaminoglycan‐PEDOT Conductive Hydrogels Facilitate Electrical Pacing of iPSC‐Derived Cardiomyocytes
Source: Adv Healthc Mater. 2025 Feb 28;14(9):2403995. doi: 10.1002/adhm.202403995 (PMC11973950; doi:10.1002/adhm.202403995)
Supplement: Supplementary file 1 — Supporting Information [file ADHM-14-0-s003.pdf]

ADVANCED HEALTHCARE MATERIALS

Supporting Information

for *Adv. Healthcare Mater.*, DOI 10.1002/adhm.202403995

Self-Doped and Biodegradable Glycosaminoglycan-PEDOT Conductive Hydrogels Facilitate Electrical Pacing of iPSC-Derived Cardiomyocytes

*Daniel Hachim, Olivia Hernández-Cruz, James E. J. Foote, Richard Wang, Matthew W. Delahaye, Daniel J. Stuckey, Zhiping Feng, Jonathan P. Wojciechowski, Luke C. B. Salter, Junliang Lin, Sian E. Harding and Molly M. Stevens**

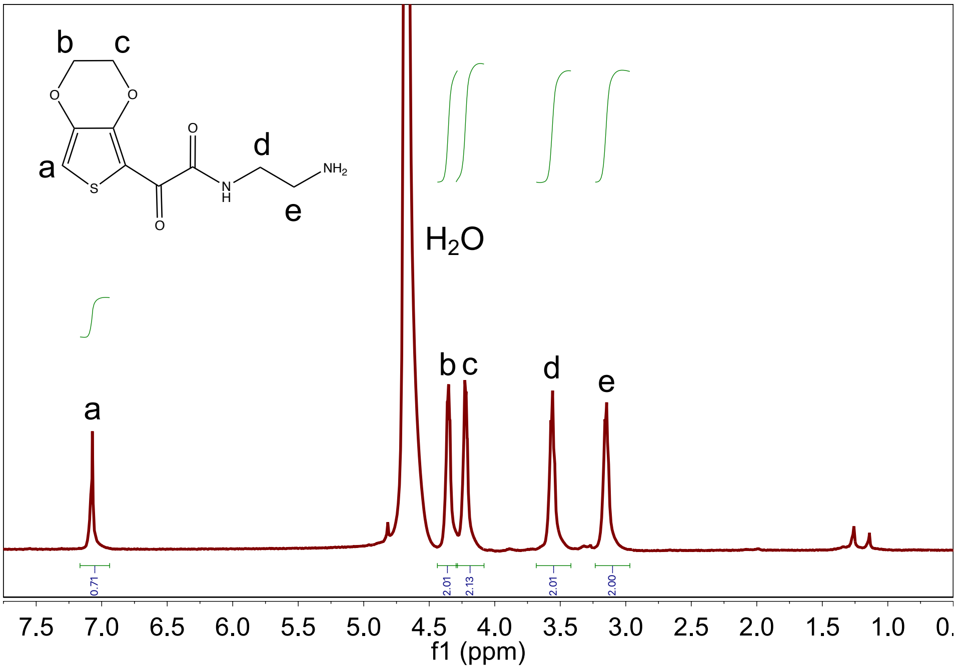

Figure S1. H[1] NMR spectrum of EDOT-NH_2 monomer in D_2O at 400 Mhz. 1H NMR (400 MHz, D2O) δ 7.19 (1H), 4.48 (2H), 4.35 (2H), 3.68 (2H), 3.27 (2H).

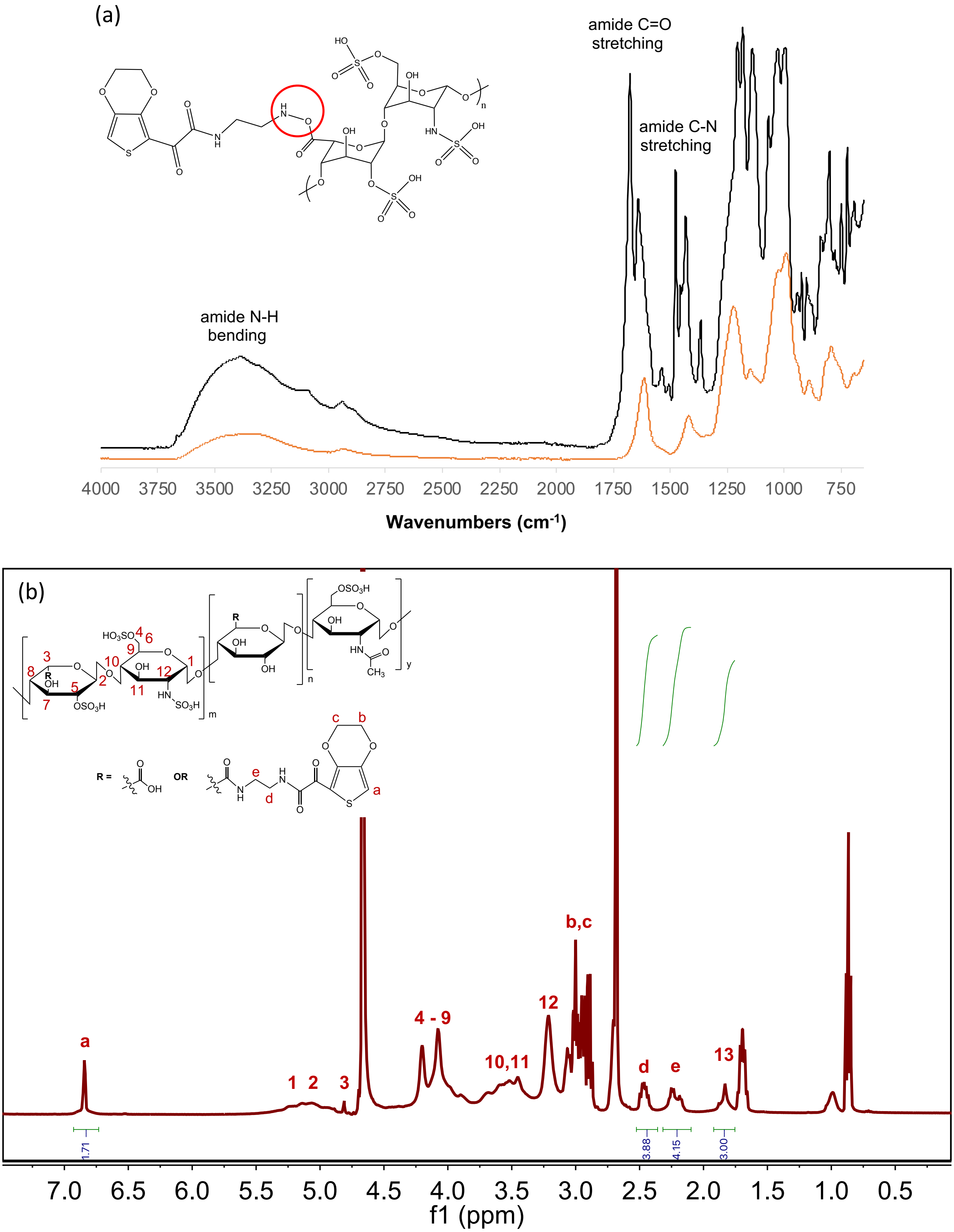

Figure S2. (a) ATR-FTIR spectrum of heparin-EDOT (black) and heparin (orange). **(b)** H^1 NMR spectrum of heparin-EDOT in D_2O at 400 Mhz.

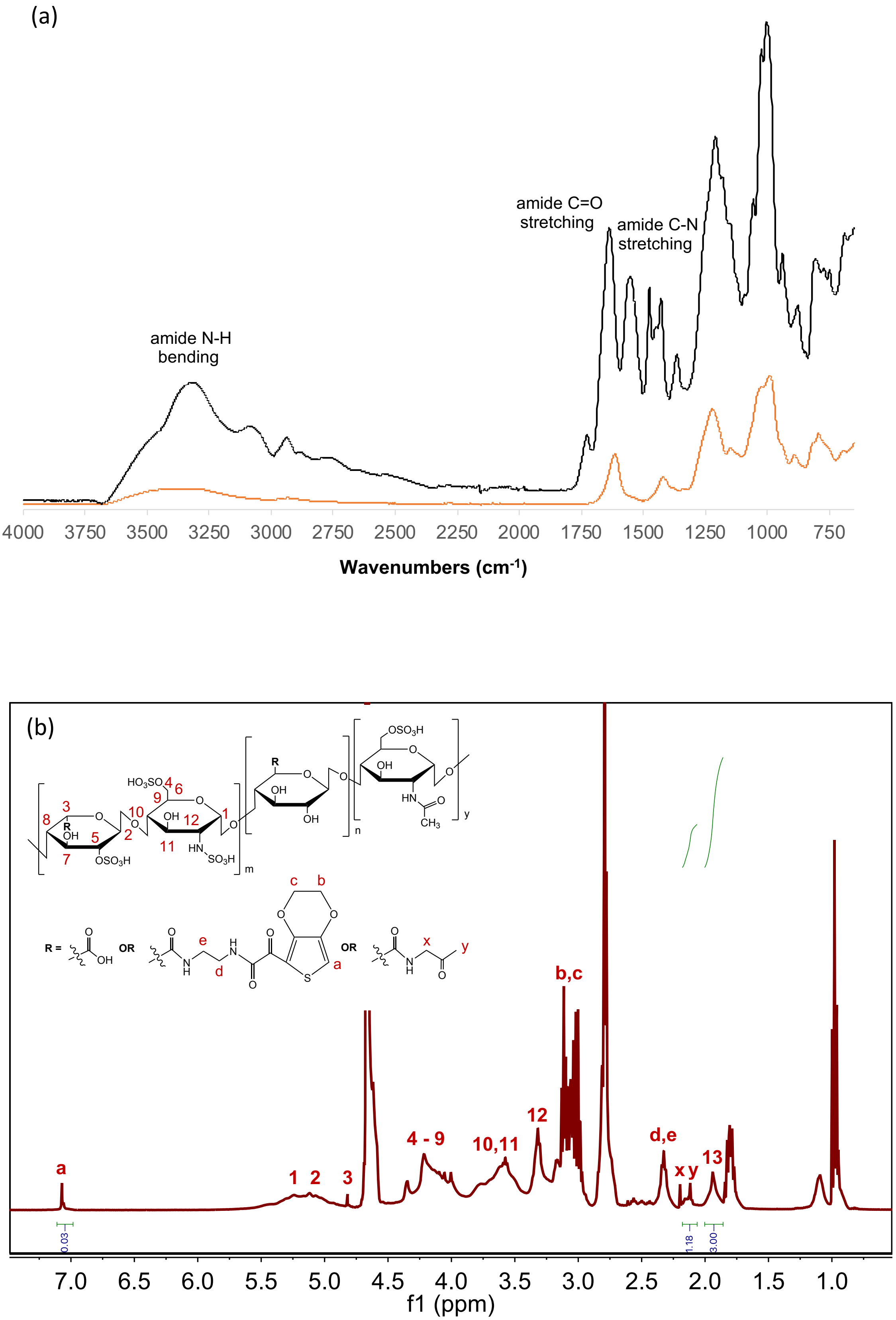

Figure S3. (a) ATR-FTIR spectrum of heparin-ketone-EDOT (black) and heparin (orange). **(b)** H^1 NMR spectrum of heparin-ketone-EDOT in D_2O at 400 Mhz.

(a)

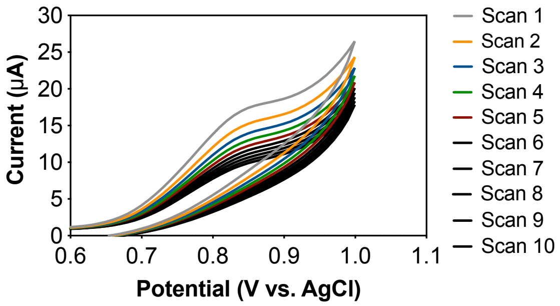

(b)

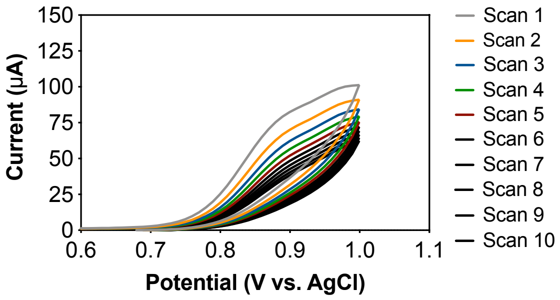

Figure S4. Electrochemical stability of HepK-PEDOT (a) and HepK-PEDOT ION (b) after consecutive cyclic voltammograms (CV) at 50 mV/s.

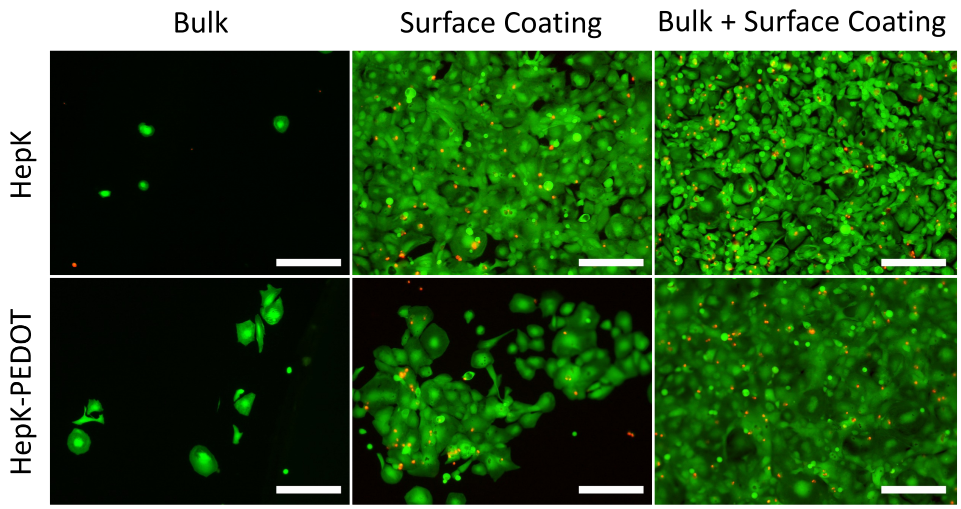

Figure S5. Fluorescence microscope images of iPSC-cardiomyocytes seeded after 48 hours on the surface of HepK and HepK-PEDOT hydrogels, in which fibronectin was incorporated in the bulk of the hydrogel during fabrication, surface coated or both simultaneously to promote cell adhesion. Live cells are shown in green (calcein) and dead cells in red (ethidium homodimer). Scale bars represent 200 µm.

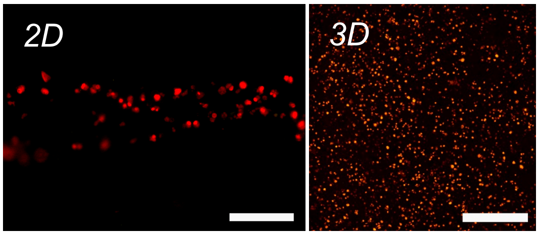

Figure S6. Fluorescence microscope images of iPSC-cardiomyocytes seeded on the surface or encapsulated within the matrix of HepK-PEDOT hydrogels with the polymer treated with ion exchange columns (ION), without pH adjustment. Live cells are shown in green (calcein) and dead cells in red (ethidium homodimer). Scale bars represent 200 µm.

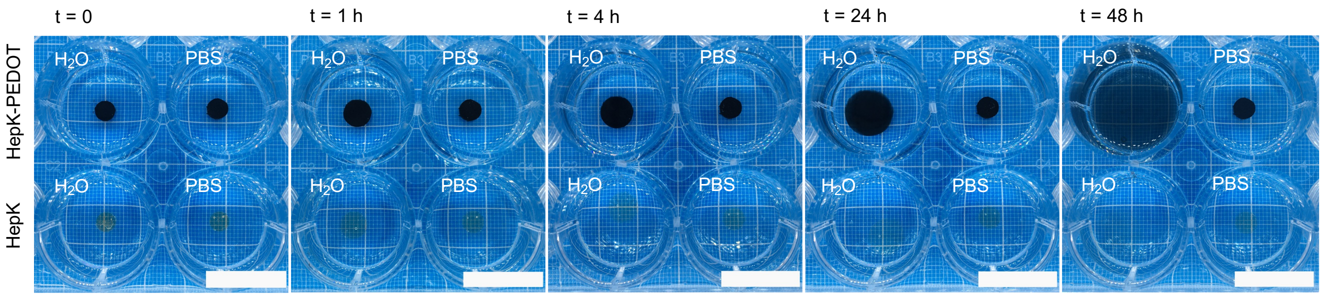

Figure S7. Stability of HepK-PEDOT and HepK hydrogels (10% total mass) incubated in H2O or 1X PBS at 37ºC for 48 hours. Scale bars represent 20 mm.

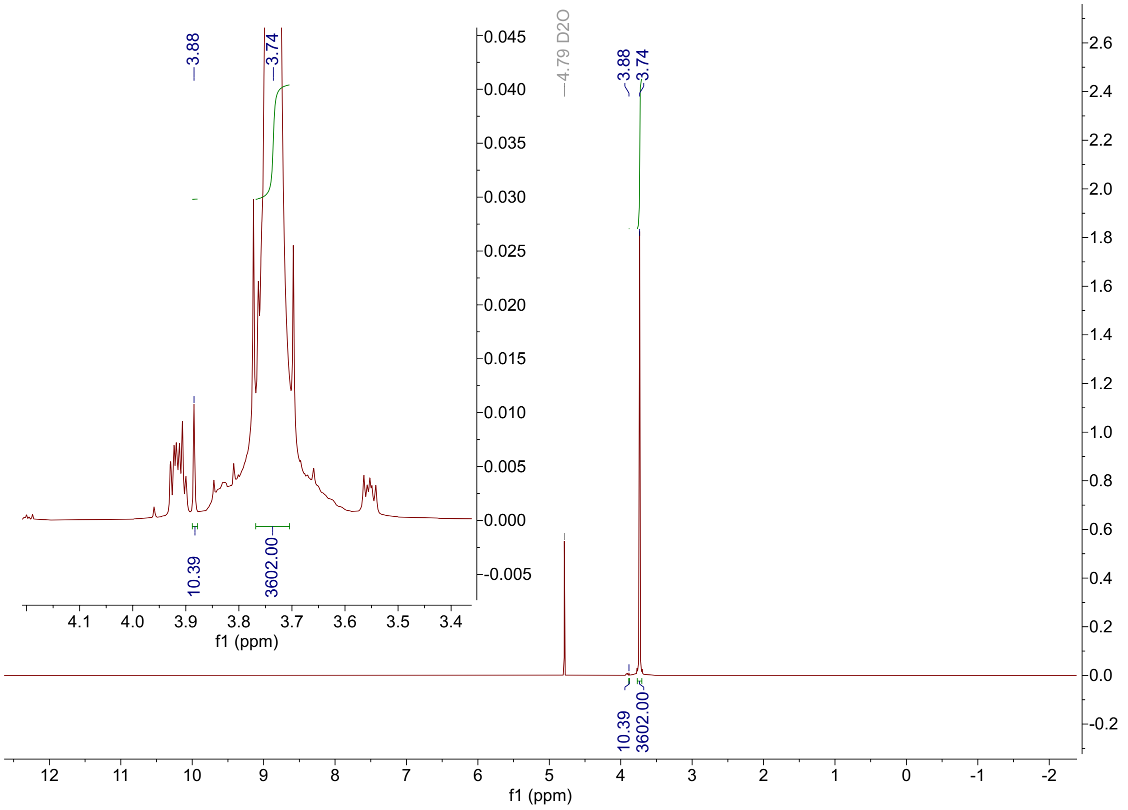

Figure S8. H[1] NMR spectrum of 8-Arm PEG-O-NH_2 in D_2O at 400 Mhz. Degree of functionalization determined to be approximately 65% based on comparing the integral at d = 3.88 ppm (NH_2-O-C$\mathbf{H_2}$-) to the integral at d = 3.74 ppm (PEG-C$\mathbf{H_2}$-).
