# Supplementary material for: Self‐Doped and Biodegradable Glycosaminoglycan‐PEDOT Conductive Hydrogels Facilitate Electrical Pacing of iPSC‐Derived Cardiomyocytes
Source: Adv Healthc Mater. 2025 Feb 28;14(9):2403995. doi: 10.1002/adhm.202403995 (PMC11973950; doi:10.1002/adhm.202403995)
Supplement: Supplementary file 2 — Description of Supplementary Videos [file ADHM-14-0-s006.pdf]

ADVANCED
HEALTHCARE
MATERIALS

Supporting Information

for *Adv. Healthcare Mater.*, DOI 10.1002/adhm.202403995

Self-Doped and Biodegradable Glycosaminoglycan-PEDOT Conductive Hydrogels Facilitate Electrical Pacing of iPSC-Derived Cardiomyocytes

*Daniel Hachim, Olivia Hernández-Cruz, James E. J. Foote, Richard Wang, Matthew W. Delahaye, Daniel J. Stuckey, Zhiping Feng, Jonathan P. Wojciechowski, Luke C. B. Salter, Junliang Lin, Sian E. Harding and Molly M. Stevens**

Description of Supplementary Videos: Representative videos of electrostimulated GCamPs-iPSC cardiomyocytes from each hydrogel group and baseline (before electrostimulation).

- **Supplementary Video 1**: HepK-PEDOT hydrogel, horizontal configuration. Pulse duration: 20ms, voltage: 5V, frequency 1HZ.
- **Supplementary Video 2**: HepK-PEDOT hydrogel, horizontal configuration. Baseline, no electrical stimulation.
- **Supplementary Video 3**: HepK-PEDOT hydrogel, vertical configuration. Pulse duration: 20ms, voltage: 4V, frequency 1HZ.
- **Supplementary Video 4**: HepK-PEDOT hydrogel, vertical configuration. Baseline, no electrical stimulation.
- **Supplementary Video 5**: HepK hydrogel, horizontal configuration. Pulse duration: 20ms, voltage: 6V, frequency 1HZ.
- **Supplementary Video 6**: HepK hydrogel, horizontal configuration. Baseline, no electrical stimulation.
- **Supplementary Video 7**: HepK hydrogel, vertical configuration. Pulse duration: 20ms, voltage: 6V, frequency 1HZ.
- **Supplementary Video 8**: HepK hydrogel, vertical configuration. Baseline, no electrical stimulation.
- **Supplementary Video 9**: HepK-PEDOT ION hydrogel, horizontal configuration. Pulse duration: 20ms, voltage: 4.5V, frequency 1HZ.
- **Supplementary Video 10**: HepK-PEDOT ION hydrogel, horizontal configuration. Baseline, no electrical stimulation.
- **Supplementary Video 11**: HepK-PEDOT ION hydrogel, vertical configuration. Pulse duration: 20ms, voltage: 2V, frequency 1HZ.
- **Supplementary Video 12**: HepK-PEDOT ION hydrogel, vertical configuration. Baseline, no electrical stimulation.
